# A fuzzy clustering approach to understanding religious and socioeconomic determinants of public perceptions of environmental regulation in the United States

**DOI:** 10.1371/journal.pone.0334868

**Published:** 2026-08-07

**Authors:** Juan Carlos Martín, Alessandro Indelicato

**Affiliations:** 1 Department of Applied Economic Analysis, Universidad de Las Palmas de Gran Canaria, Las Palmas de Gran Canaria, Spain; 2 Universidade de Vigo, Vigo, Spain; The University of Warwick, UNITED KINGDOM OF GREAT BRITAIN AND NORTHERN IRELAND

## Abstract

The study examines the socio-economic, ideological and religious factors influencing how likely Americans perceive the consequences of environmental regulation to be. Three perception-based items from the Pew Research Center’s American Trends Panel Wave 106 (n = 10,156) are examined: higher fuel and electricity prices, gradual loss of individual freedoms, and fewer jobs or declining pay in fossil-fuel-dependent industries. These items are interpreted as subjective expectations rather than observed or causal effects of regulation. We use fuzzy hybrid TOPSIS to create a synthetic index of the perceived effects of environmental regulation on Americans (AP-ERE), and fuzzy clustering to classify respondents as extremely convinced, extremely unconvinced or intermediate. The results reveal significant disparities among ideological, socioeconomic, and religious groups. The convinced cluster is more prevalent among religious, conservative, older and male respondents, suggesting that they perceive a greater likelihood of economic and social costs. Overall, the results suggest that perceptions of environmental regulation are shaped not only by ideology and socio-economic characteristics, but also by religious identity and religiosity.

## Introduction

Global warming, climate change and environmental concerns have become increasingly prominent issues on the international public agenda. However, as these issues constitute negative externalities that the market does not adequately address, governments have been compelled to intervene through environmental regulations. The intersection of environmental problems and institutional responses has sparked growing interest in the factors influencing citizens’ attitudes towards such regulations. Recent studies, such as those by El Deeb et al. [1] and Villaseñor-Ibáñez et al. [2], have analysed voting trends and the influence of religious and socio-economic factors on public opinion in the United States. This is fundamental for designing more effective and widely accepted environmental policies.

Adelle et al. [3] argued that growing awareness of these externalities led Europe and the United States to first implement local environmental policies, and subsequently more transnational regulations. This shaped a new agenda for policymakers and international diplomats. However, environmental regulations are not exempt from citizens’ concerns and criticism. According to the Pew Research Center [4], for example, ‘many religious Americans voice concerns about the potential consequences of environmental regulations, such as a loss of individual freedoms, fewer jobs, or higher energy prices’ (p. 7).

The issue of climate change gained significant attention when scientists, for example, proved that the ozone layer was becoming thinner in some parts of the world, causing an increase in skin cancers due to emissions from highly industrialised countries. In 1988, the Intergovernmental Panel on Climate Change (IPCC) was established, followed by the United Nations Framework Convention on Climate Change in 1992. These institutions are responsible for establishing an overarching framework for global cooperation to address climate change, recognising the need to stabilise greenhouse gas concentrations in the atmosphere, and assessing the scientific basis of climate change to guide policymakers. Subsequently, various climate treaties, such as the Kyoto Protocol 1997 and the Paris Agreement 2015, were signed by some countries to raise awareness of climate change, promote international cooperation, and encourage action to limit its effects.

Pew Research Center [4] found that a significant portion of the sample (42%) acknowledges that the impact of environmental regulations on the economy will cause a gradual loss of individual freedom, with Evangelical Protestants (56%), Protestants (48%) holding this view more intensively than non-religious affiliated citizens (34%). A similar pattern is also found in the impact that environmental regulations could have on the economy. About half of Americans who affiliate with a religion say that stricter environmental laws and regulations will cost too many jobs and hurt the economy. Again, the Evangelical Protestants are especially likely to hold this view. Indeed, they are the only major U.S. religious group in which a majority (66%) take this position.

The study conceptually differentiates between the objective effects of environmental regulation and the subjective perception of those effects. The empirical indicators used in the analysis do not measure real changes in energy prices, employment levels or individual freedoms. Instead, they capture respondents’ expectations of how likely these outcomes are to occur due to environmental regulation over the next 30 years. This distinction is important, as energy prices, labour market outcomes, and perceived restrictions on individual freedom are shaped by a range of mechanisms, including market dynamics, technological change, regulatory design, political ideology, and broader energy transitions.

Environmental regulation is rooted in the urgent and clear challenge that the earth faces: “How do we maintain or improve the quality of life for the planet’s inhabitants while ameliorating the harm already done and preventing future harm to the environment? One essential part of the strategy has to be to stop, on a global net basis, emitting gases to the atmosphere that warm the Earth, especially but not exclusively carbon dioxide, because of its relatively high concentration and long life in the atmosphere [5] (p. viii)”.

However, environmental regulation in the form of a global transition to net-zero greenhouse emissions will require innovation, technological progress, and some societal costs regarding electricity prices, loss of freedom, lack of economic growth, or a substantial change in the way society produces the necessary energy that will be associated with a complete change in the human lifestyle.

The aim of the paper is fivefold. First, we construct a synthetic indicator of the perceived effects of environmental regulation on Americans (AP-ERE) using a fuzzy hybrid TOPSIS approach. Second, we identify the ideal solutions of the three perception-based items that comprise the latent construct. Third, we explore the variation in AP-ERE across different socioeconomic, ideological, and religious groups. Fourth, we apply fuzzy clustering to classify respondents according to their degree of membership in the convinced, unconvinced, and intermediate perception profiles. Fifth, we test the association between religious affiliation, religiosity, and religious attendance and different cluster membership weights.

The remainder of the paper is organised as follows: Section 2 offers insights from the literature, Section 3 describes the data, Section 4 details the methodology, Section 5 presents and discusses the results, and Section 6 offers concluding remarks.

## Literature review

### Environmental regulation impacts

National Academies of Sciences, Engineering, and Medicine [5] contended that the highest priority action to mitigate CO2 emissions is decarbonising electric power and eliminating fossil-fuel combustion sources. A second action will also eliminate the use of fossil fuels from the transport industry and natural gas for heating. The remaining measures will occur via carbon management using innovative chemistry components.

Environmental regulation aimed at reducing greenhouse gas emissions is often associated with rising costs, such as electricity costs [6]. However, it is important to consider [7] hypothesis that well-designed environmental regulation can stimulate technological innovation and efficiency. This can lead to reduced costs and improved long-term competitiveness [7]. López-Gamero et al. [8] also highlight the potential of environmental regulation to transform managerial perception for other renewable energy sources like windmills or photovoltaic plants. The technology of solar and wind farms is still relatively new and expensive compared to the other energy sources, and the plants require a lot of land and infrastructure [9]. The new sources are also intermittent, producing electricity inconsistently either when the sun is shining or the wind is blowing, making it uncertain to match supply and demand [10]. Renewable energy plants need to be integrated into the existing electricity grid, which can be expensive because the grid needs to handle the intermittent nature of renewable energy sources. Renewable energy sources cannot be stored easily, so it is necessary to have storage facilities in place to store excess electricity when it is not needed, and these facilities are expensive and inefficient [11]. Moreover, finally, renewable energy plants are often located in remote areas, far from where the electricity is needed. Electricity needs to be transmitted over long distances [12].

Environmental regulation can lead to higher compliance costs, but it can also foster innovation and, through the development of cleaner technologies, partly offset these costs [7,13]. Fuel and electricity prices are also determined by other mechanisms, such as supply-and-demand conditions, wholesale market design, energy market volatility, infrastructure constraints, geopolitical shocks, technological costs, and policy design [14,15,16].

The relationship between environmental regulation and human freedom is complex and contested. There are valid arguments to be made on both sides of the issue. Ultimately, deciding how to balance environmental protection with individual freedom is a matter of political judgment. There are several reasons why some people might assume that environmental regulation affects human freedom. One reason is that environmental regulations can sometimes restrict the activities of businesses or individuals. For example, regulations that protect endangered species or sensitive ecosystems can restrict the development of land or the use of natural resources [17,18]. Another reason for the belief that environmental regulation is incompatible with human freedom is that some individuals see governmental intervention in the economy and private lives of individuals as absolutely unacceptable. Some individuals may prefer more control over their own environmental decisions rather than having them dictated by government mandates [19,20].

Despite these concerns, there are also strong arguments to be made that environmental regulations are compatible with human freedom. One argument is that, in the long run, environmental protection is essential for human health and well-being. For example, air and water pollution can cause serious health problems, and climate change can lead to more extreme weather events like floods, droughts, and wildfires [21,22]. Another valid argument is that environmental regulation can promote economic growth and innovation. For example, clean energy technologies can create new jobs and industries, and regulations that promote sustainable practices can make businesses more efficient and productive [23,24].

Environmental regulations usually clash over the need to maintain the level of employment in the fossil fuel industry. This has been especially seen in the coal industry in some of the most industrialised countries in the world. Environmental regulations have contributed to the decline of the coal industry worldwide. However, it is crucial to recognise that this decline cannot be attributed solely to environmental regulations. Other significant factors, such as the growing availability and competitiveness of natural gas, have played a key role in reshaping the energy landscape [25], including mining, transportation, and power generation. The magnitude of these job losses varies across regions and countries, depending on the specific regulations and the reliance on coal as an energy source. The global coal employment has declined by 20% since 2012, with the most significant decline in China. The United States has seen a significant decline in coal mining employment, with job losses estimated at over 50,000 since 2008. In Europe, the coal industry has also faced job losses due to environmental regulations, particularly in Germany, where the government has implemented policies to phase out coal-fired power plants. [26, 27].

### Environmental regulation effects surveys

Environmental regulation is considered a fundamental tool of the overall climate change policy to improve energy efficiency and mitigate environmental problems caused by greenhouse gas emissions. Shahzad [28] provides a detailed survey of the interaction between environmental regulation, energy consumption and environmental quality of the earlier literature up to 2020 for developed, developing, and emerging countries. The study mainly focused on three types of causality direction: (i) environmental taxes, energy consumption, and energy efficiency; (ii) environmental taxes and environmental quality; (iii) energy consumption (renewables, non-renewable, and fossil fuels) and environment deterioration. The author concluded that the role of environmental regulation in the form of taxes is still ambiguous and demands a more in-depth investigation, but, in general, environmental taxes mitigate greenhouse gas emissions by discouraging the use of fossil fuels.

National Academies of Sciences, Engineering, and Medicine [29] contended that environmental regulation is usually needed in those communities that bear the most significant burden and that the measures should address very complex problems that are multidisciplinary, of global relevance, and whose impacts are caused by multiple intersecting factors. Environmental regulation measures can be classified into four different categories such as command and control or administrative supervision, market or economic incentive (introducing some Pigouvian tax), public participation or public legislation, and voluntary action [30,31]. Environmental regulation effects have been analysed through a prism of regional and industrial economics using three main theoretical backgrounds, namely “Porter”, “Pollution Haven”, and “Race-to-the-bottom” [31].

We were unable to find any established scale designed to measure perceptions of the effects of environmental regulation. However, there is a well-established body of literature on the perception of regulation. Braithwaite’s [32] work, for example, introduces the concept of motivational stances to explain how individuals and organisations respond to regulation [32]. However, during the search, some studies dealt with the pro-environmental behaviour scale [33,34,35,36]. Markle [33] reviewed 49 studies measuring pro-environmental behaviour and found that various proposed measures lacked consistency. The author proposed 19 items in four dimensions: conservation, environmental citizenship (activist), food and transportation. The author validated the scale, including government environmental regulation support that protects environmental quality. Menardo et al. [34] adapted the previous scale to the Italian case, finding the same dimensions but only 15 items.

Ogunbode et al. [35] included the following eight items to measure pro-environmental behaviour (p.6): (1) cycle or walk instead of driving, (2) restrain oneself from buying unneeded new clothes, (3) choose not to fly, (4) try to influence family and friends to act pro-environmentally, (5) save energy in the household, (6) take public transportation instead of the car, (7) avoid food waste, and (8) make climate-friendly food choices. Responses to the items were rated in an anchored 5-point semantic scale between “1 = almost never” and “5 = almost always”.

Whitmarsh et al. [36] also included eight items by asking (p. 4), ‘At the moment, roughly how many times per month do you do each of the following?‘: Eat organic, locally-grown or in-season food; Encourage other people to save energy; Buy products with less packaging; Recycle household waste (e.g., glass); Avoid wasting food (e.g., by using leftovers); Buy second-hand items; Borrow or rent items (e.g., tools, toys); and Repurpose something for a different use, instead of throwing it away. The answer format was based on a 7-point anchored scale from ‘not at all’ (1) to ‘at least once a day’ (7).

In both scales, all the actions involve a relatively low environmental impact, which means that higher figures are aligned with more pro-environmental behaviour. Interestingly, for the aim of the current study, most of the items included in the scales are related to an apparent loss of consumers’ freedom regarding choices in transport, food, clothes and energy. Here, the gradual loss of individual freedoms is not considered a directly observable institutional outcome, but rather a subjective evaluation of potential regulatory overreach. Previous research on the acceptability of climate and energy policy indicates that citizens’ support for environmental measures depends on a variety of factors, including expected environmental benefits, perceived personal costs, fairness, trust, and the extent to which policies interfere with everyday behaviour [37,38,39]. Consequently, respondents may associate environmental regulation with perceived limitations on mobility, consumption, land use, energy choices, or lifestyle preferences. The item thus draws on perceptions of constraint rather than on objective losses of individual freedom.

The second scale also introduces some items related to the circular economy or the conservation dimension. Thus, it can be concluded that the perception of environmental regulation effects has not been measured, and this is the first attempt in the literature. The scale is based on the PEW questionnaire that uses only three items measuring how likely some events will happen within the next 30 years because of environmental regulation. Although earlier research has explored public attitudes towards environmental protection, none has directly conceptualised a scale to measure the perceived effects of environmental regulation itself. Traditional regulatory perception research, such as Braithwaite’s [32] work on motivational postures, has examined compliance and trust in authorities, but not expectations of environmental policy outcomes. Therefore, our three Pew-based items differ conceptually in that they capture expectations of specific potential consequences attributed to environmental regulation rather than general compliance attitudes.

### Data

#### Survey and Respondents.

A survey conducted by the Pew Research Center aimed to explore the relationship between American’s religious beliefs and their views on climate change and the environment. The survey was conducted from April 11 to April 17, 2022, and involved a nationally representative sample of adults aged 18 years and older (n = 10,156) across the US, including Alaska and Hawaii. The respondents came from the American Trends Panel (ATP), formed through national random sampling of residential addresses. Participants completed self-administered web surveys, and those without internet access received tablets and wireless connections from Ipsos, the panel’s managing company. Interviews were conducted in English and Spanish, with the Pew Research Center developing the questionnaire in collaboration with Ipsos. The margin of sampling error is plus or minus 1.6 percentage points, and participants received incentives of $5 to $20 based on response tendencies. More details are available in the study’s technical report [4].

#### Variables.

Three questions in the survey are used as items of the AP-ERE latent variable. The three items were included in the variable named (GOVWRRY), for which respondents were asked, “How likely do you think each of the following is to happen within the NEXT 30 YEARS because of environmental regulations? The three items were randomised to minimise the potential biased responses and correspond to the following wordings: (1) Much higher prices for fuel and electricity; (2) Gradual loss of individual freedoms; and (3) Fewer jobs and declining pay in industries that depend of fossil fuels. The answer format options for each item were based on a full 5-point semantic scale: 1 Extremely likely, 2 Very likely, 3 Somewhat likely, 4 Not too likely, and 5 Not at all likely. According to the respondents’ perception, the original response scale was reverse-coded so that higher values corresponded to a higher perceived likelihood of the corresponding consequence occurring. The item on higher fuel and electricity prices is treated in this study as a perceived regulatory consequence rather than an objective estimate of the price effect of regulation.

The three GOVWRRY items were operationalised as ordinal indicators of perception. The empirical construct measures perceived likelihood rather than observed regulatory impact, as the survey question asks respondents to report how likely they think each outcome is to occur. The AP-ERE latent construct should thus be interpreted as a synthetic measure of subjective expectations about the effects of environmental regulation.

The following eleven socioeconomic and demographic variables are included in the analysis: language, life in the next ten years, the relative importance of CC as a problem, the relative importance of energy conservation, the self-assessment of being a religious person, age, gender, religion, religious service attendance, ideology and income. The aim of the paper makes that some variables associated with religious and religious practices need to be selected. Religious affiliation was analysed using the disaggregated categories available in the Pew data set. In the estimation procedure, no religious categories were combined. Where the narrative interpretation discusses several groups together, this reflects similarities in descriptive patterns or membership weights, but not as a formal aggregation in the empirical model.

#### Methodology.

Fuzzy set methods were first introduced by Zadeh [40] as a novel method for addressing vague, subjectively judged information provided by respondents. In these cases, the p crisp categories 1-p, provided by Likert or semantic scales are better represented through fuzzy logic sets [41,42], is assigned a membership value in [0, 1] and it is known as the intensity or degree of belonging information. In contrast to other traditional methods, fuzzy methods provide a practical approach to dealing with the inherent imprecision of responses [43,44,45,46,47]. This analytical approach enables us to represent respondents’ subjective judgements as perceived likelihood, which is consistent with the perceptual rather than causal nature of the study’s research question.

The dataset for the primary AP-ERE latent variable is derived from the responses of panellists using a 5-point semantic scale, making fuzzy-hybrid multi-criteria decision-making (MCDM) and fuzzy clustering methods well-suited for this analysis [41,48,49]. The fuzzy set methods have gained immense popularity in various specialised fields.

Zadeh [50] defined a linguistic variable as an answer expressed in natural language. He introduced fuzzy sets as an application to approximate reasoning, where the universe of discourse and associations are two crucial concept components. Different universes and associations have been proposed in the literature [43,51]. This study uses triangular fuzzy numbers (TFNs) as in Leon & Martin [52], the following association is established: Not at all likely (0, 0, 30), Not too likely (20, 30, 40), Somewhat likely (30, 50, 70), Very likely (60, 70, 80) and Extremely likely (70, 100, 100).

In the study, we use the centroid method proposed by Chen [53] to calculate the defuzzified value of the average TFN of each of the groups of population of interest. This method is still broadly used in the literature because it is resilient and not influenced by researchers’ optimistic or pessimistic judgments [54]. Kumar [55] recently demonstrated that the total integral value is equivalent to it.

We apply the fuzzy-hybrid approach to the defuzzified matrix to calculate the AP-ERE indicator that measures the perception of Americans of the environmental regulation effects. The indicator determines which segment is more or less convinced about the occurrence of environmental regulation effects within the next 30 years. A particular segment is more convinced of the studied effects whenever the relative index is closer to 1. Interested readers can consult previous studies to find all the steps needed to calculate the indicators [52].

The AP-ERE index is based on ordinal subjective assessments that are transformed using triangular fuzzy numbers and subsequently defuzzified. The index does not measure exposure to environmental regulation or the observed consequences of policy. Instead, it ranks respondents according to the perceived strength of the likelihood that the three regulation-related outcomes will occur.

Finally, we apply the fuzzy clustering method following the three-cluster solution considered by D’Urso et al. [56], named as: (1) extremely convinced, extremely unconvinced, and intermediate convinced respondents. Each respondent’s profile is selected according to the maximum, minimum, and median of the individual respondent’s synthetic AP-ERE. For ease of exposition, we omitted the mathematical formulation of the method, but interested readers can consult [57,58,56] and Indelicato and Martín [59].

## Results

Table 1 shows the ideal solutions, the representative population segment for each item, and the percentage variation between both ideal solutions. It can be seen that the representative segments for both ideal solutions are highly concentrated in two particular population groups. Those who consider it extremely likely that the United States will create many unnecessary environmental regulations, that is, they think the country is regulating the environment very tight and those who consider this as an extreme necessity. The only exception in A- is observed in the item referred to as “the much higher prices for fuel and electricity”, represented by those who did not answer whether they were eating food that requires a lot of production energy, as this fact is only known for a well-informed segment or because the food labels provide that type of information. We will provide more insights in the discussion section. Regarding the last column of the table, it can be seen that respondents show the most heterogeneous answers for the item “gradual loss of individual freedom”.

**Table 1:** Fuzzy Hybrid TOPSIS Ideal Solutions.

Item	A^+^	A ^+^ . Rep.	A^-^	A^-^. Rep.	Perc.Var.
Much higher prices for fuel and electricity	86.00	(*)	59.68	(-)	44.1%
Gradual loss of individual freedom	81.00	(*)	34.34	(--)	135.9%
Fewer jobs and declining pay in industries that depend on fossil fuels	77.54	(*)	55.11	(--)	40.7%

(*)The United States will create many unnecessary environmental regulations. Extremely likely.

(-)Eating food that requires a lot of production energy. (NA).

(--)The United States will create many unnecessary environmental regulations. Not at all likely.

The interpretation of the ideal solutions must be limited to the perceptual scale used in the study. Higher values imply more positive subjective expectations that the three potential consequences will happen. They should not be taken as evidence of the objective effects of environmental regulation on prices, employment, or individual freedoms.

It is out of the scope of the current study to focus on the results of the AP-ERE synthetic index. However, as a descriptive result, we find higher convinced membership weights among respondents who think that life in 10 years will be worse, do not perceive climate change or energy conservation as serious problems, are very religious, belong to older age groups, are male, attend religious services more than once a week, are very conservative, and belong to the middle-income class. Among religious affiliates, Protestant respondents have the highest average convinced membership weight, while Mormon/Latter-day Saint respondents have the second highest. In the empirical analysis, these categories are reported separately and are not formally pooled.

The fuzzy hybrid TOPSIS method was also used to find the three representative profiles of each cluster. The convinced representative respondent answered with 5s to the three items, the unconvinced representative respondent answered with 1s to the three items, and the intermediate respondent answered with 4s to the first and third items and a 3 to the second item of the LV scale.

**Fig 1:**
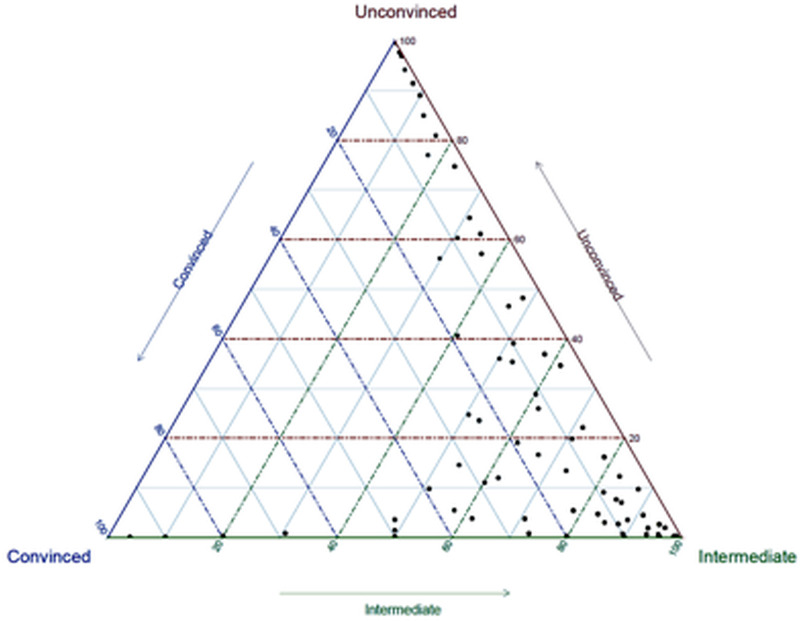
Fuzzy clustering ternary graph.

Fig 1 shows the ternary plot for the whole sample. The figure shows that there is considerably more heterogeneity in the group of respondents who are more similar to the intermediate profile than for the rest of the clusters, namely convinced and unconvinced citizens.

The average probability of belonging to each cluster shows that the “convinced,” “unconvinced,” and “intermediate” clusters are represented by 30.8, 7.5, and 61.7 per cent, respectively. Thus, it can be concluded that most citizens perceive environmental regulations as likely to be associated with higher fuel and electricity prices, a gradual loss of individual freedoms, and fewer jobs or declining pay in industries that depend on fossil fuels. This finding should be interpreted as the respondents’ perceived likelihood, not as evidence of actual regulatory effects.

Table 2 shows the ANOVA performed for the convinced weights using the eleven covariates under analysis. The table shows whether there are significant differences in the convinced weight obtained after applying the Fuzzy Clustering. The table shows the average values, the standard deviation, and the discussion of whether there are significant differences according to whether the average of some categories is significantly lower than those marked through the respective superindex. For brevity and concision, the table shows only those segmentation variables of interest for which significant statistical differences are obtained. Thus, it can be seen that language or income does not appear in the table because no significant differences were obtained.

**Table 2:** One-way ANOVA Analysis for convinced weights.

Name	Average	SD	Disc.
Life in 10 years better	0.239	0.006	1-2
Life in 10 years about the same¹	0.264	0.006	2
Life in 10 years worse²	0.498	0.008	
Climate Change is a very serious problem	0.181	0.007	2-4
Climate Change is a extremely serious problem¹	0.192	0.006	2-4
Climate Change is somewhat serious problem²	0.310	0.008	3-4
Climate Change is not too serious problem³	0.542	0.009	4
Climate Change is not a problem⁴	0.682	0.012	
Energy conservation is a very serious problem	0.245	0.007	1-4
Energy conservation is a extremely serious problem¹	0.294	0.009	3-4
Energy conservation is somewhat serious problem²	0.309	0.006	3-4
Energy conservation is not too serious problem³	0.459	0.012	4
Energy conservation is not a problem⁴	0.619	0.023	
I am not at all religious	0.222	0.008	1-3
I am not too religious¹	0.276	0.008	2-3
I am somewhat religious²	0.331	0.006	3
I am very religious³	0.406	0.009	
Age. 18–29	0.210	0.013	1-3
Age. 30–49¹	0.282	0.007	2-3
Age. 50–64²	0.337	0.007	
Age. 65 + ³	0.338	0.007	
Female	0.283	0.005	1
Male¹	0.340	0.006	
Atheist	0.186	0.016	6, 9-11
Agnostic¹	0.202	0.016	9-11
Muslim²	0.206	0.050	
Buddhist³	0.227	0.047	
Jewish⁴	0.230	0.027	11
Orthodox^5^	0.235	0.053	
Religion. Nothing in particular^6^	0.255	0.010	9,11
Hindu⁷	0.256	0.055	
Other religion⁸	0.276	0.025	11
Roman Catholic⁹	0.310	0.008	11
Mormon/ Latter-day Saint^10^	0.351	0.027	
Protestant¹¹	0.368	0.006	
Attendance. Never	0.260	0.008	3-5
Attendance. Once or twice a month¹	0.277	0.014	4-5
Attendance. Seldom²	0.291	0.008	4-5
Attendance. A few times a year³	0.314	0.010	5
Attendance. Once a week⁴	0.353	0.009	5
Attendance. More than once a week^5^	0.431	0.014	
Liberal	0.142	0.009	2-4
Very liberal¹	0.155	0.013	2-4
Moderate²	0.237	0.006	3-4
Conservative³	0.450	0.007	4
Very conservative⁴	0.597	0.011	

The discussion column shows the statistical differences observed for each of the covariates that present some significant differences. Thus, in the first row of life in ten years will be better, the column shows 1–2, meaning that the average of the convinced weights 0.239 is statistically lower than the average of the groups which consider that life in ten years will be about the same (0.264) and will be worse (0.498). Thus, it seems that those who have a very pessimistic opinion about their future life are more convinced than the rest of the citizens about the effects of environmental regulation effects. All the results will be commented on in the next section.

The results of the ANOVA show that only two covariates, namely language and income, do not affect the convinced citizens cluster. Some significant differences were obtained for the rest of the nine covariates included in the study. It is interesting to remark that the three religious variables included have some effect.

In a nutshell, the study concludes that belonging to the convinced cluster is higher for: (1) those who consider life in ten years will be worse; (2) climate change is not a problem; (3) energy conservation is not a problem; (4) very religious citizens; (5) older generations; (6) males; (7) Mormons and Protestants; (8) attend more than once a week to religious services; and (9) very conservative citizens.

Table 3 shows the ANOVA performed for the unconvinced weights using the eleven covariates under analysis. The table is interpreted similarly to what has been explained in Table 2.

**Table 3:** One-way ANOVA Analysis for unconvinced weights.

Name	Average	SD	Disc.
Spanish	0.0446	0.0090	1
English¹	0.0760	0.0020	
Life in 10 years worse	0.0413	0.0039	1-2
Life in 10 years about the same¹	0.0730	0.0032	2
Life in 10 years better²	0.0945	0.0029	
Climate Change is not too serious problem	0.0393	0.0050	3-4
Climate Change is not a problem¹	0.0438	0.0063	3-4
Climate Change is somewhat serious problem²	0.0559	0.0042	3-4
Climate Change is a very serious problem³	0.0812	0.0039	4
Climate Change is a extremely serious problem⁴	0.1057	0.0033	
Energy conservation is not too serious problem	0.0578	0.0060	4
Energy conservation is somewhat serious problem¹	0.0698	0.0031	4
Energy conservation is a very serious problem²	0.0769	0.0033	
Energy conservation is not a problem³	0.0846	0.0118	
Energy conservation is a extremely serious problem⁴	0.0882	0.0045	
I am very religious	0.0552	0.0043	2-3
I am somewhat religious¹	0.0601	0.0032	2-3
I am not too religious²	0.0771	0.0041	3
I am not at all religious³	0.1111	0.0039	
Age. 65+	0.0571	0.0035	1-3
Age. 50–64¹	0.0738	0.0035	3
Age. 30–49²	0.0853	0.0034	
Age. 18–29³	0.0995	0.0065	
Mormon/ Latter-day Saint	0.0335	0.0136	3, 5-7, 9-11
Protestant¹	0.0570	0.0029	3, 6-7, 9-11
Roman Catholic²	0.0685	0.0041	6, 9-11
Religion. Nothing in particular³	0.0885	0.0048	9, 11
Orthodox⁴	0.0934	0.0260	
Other religion^5^	0.0967	0.0122	
Agnostic⁶	0.1001	0.0078	
Jewish⁷	0.1120	0.0134	
Buddhist⁸	0.1216	0.0232	
Atheist⁹	0.1271	0.0080	
Muslim^10^	0.1566	0.0247	
Hindu¹¹	0.1851	0.0270	
Attendance. More than once a week	0.0495	0.0069	4-5
Attendance. Once a week¹	0.0619	0.0043	4-5
Attendance. A few times a year²	0.0643	0.0050	5
Attendance. Seldom³	0.0722	0.0038	5
Attendance. Once or twice a month⁴	0.0872	0.0070	
Attendance. Never^5^	0.0961	0.0038	
Very conservative	0.0368	0.0060	2-4
Conservative¹	0.0391	0.0036	2-4
Moderate²	0.0734	0.0031	3-4
Liberal³	0.1213	0.0045	4
Very liberal⁴	0.1443	0.0066	

Table 3 summarises the main characteristics of the citizens with higher unconvinced membership weights. These are: respondents who completed the questionnaire in English, expect life in ten years to be better, consider climate change and energy conservation to be extremely serious problems, are not at all religious, belong to younger age groups, never attend religious services and identify as very liberal. Hindu, Muslim and atheist respondents are associated with relatively larger unconvinced membership weights than several other religious-affiliation categories. These are categories which are reported separately in the empirical analysis and are not formally combined.

The results of the ANOVA show that only two covariates, namely gender and income, do not affect the unconvinced citizens cluster. Out of the nine covariates that influenced the unconvinced cluster, it was observed that there were significant differences in some of them. It was particularly noteworthy that the three religious variables included in the analysis showed some influence. This implies that religion may play a role in shaping the study’s outcome. Further research may be required to fully understand the extent of the impact of these variables on the study.

## Discussion

The ideal solutions results show a high heterogeneity of responses for the item “gradual loss of individual freedoms”, and this heterogeneity was observed for the opposite answers (extremely likely vs. not at all likely) given to the question “The United States will create many unnecessary environmental regulations”. Environmental regulations can restrict business activities, e.g., land development [17,18]. Some people believe that environmental regulations are not compatible with human freedom. They oppose governmental intervention in the economy and individual lives, preferring more control over their environmental decisions rather than having them dictated by government mandates [19,20].

These results highlight that the present analysis concerns not the objective consequences of regulation but subjective expectations about its societal impact. Respondents may project broader concerns — economic, political or ideological — onto the concept of environmental regulation. This tendency has been observed previously in studies of belief-driven policy perceptions [32,1]. Therefore, the three identified clusters should be interpreted as attitudinal profiles rather than as direct indicators of regulatory effectiveness.

Excessive and unnecessary environmental regulations may arise due to the demands of multiple stakeholders who want the Environmental Protection Agency (EPA) to demonstrate that compliance is important. This is often reflected in formal EPA policies and the vision of senior environmental officials at both state and federal levels. It is commonly assumed that most companies comply and that non-compliance is mainly observed in smaller companies. However, Giles [60] disputes this notion with a resounding “NO”.

The context of the dataset, being a recent American survey and the items included in the scale, makes comparing the obtained results a difficult task, as the scale was mainly studied as a pro-environment behaviour, which is not the same as the LV studied AP-ERE. Pro-environmental behaviour is usually governed by how the benefits and costs of the rule are tallied up. It has to be self-evident that the benefits must be higher than the costs to engage in that behaviour. However, real-life courses are not usually so self-evident.

It was shown that, on average, the probability of belonging to each cluster shows that the “convinced”, “unconvinced”, and “intermediate” clusters are represented by 30.8, 7.5 and 61.7 per cent, respectively. It was unsurprising that most of the respondents expressed mixed feelings about the effects of environmental regulation. At the same time, most citizens believe that environmental regulation will cause much higher prices of fuel and electricity in comparison with the other two items included in the scale. It is important to note that there is a wide range of views on climate change and environmental regulation. Some people believe that climate change is a very serious problem and that we must immediately address it. Others believe that climate change is a less serious problem or that the costs of taking action outweigh the risks of climate change. Similarly, there is a wide range of views on the effectiveness and efficiency of environmental regulations. In Heyvaert’s [61] words, “the study of environmental law and regulation is a rewarding but emotionally draining enterprise (p. xi)”.

The results obtained for the intersection of the convinced and unconvinced cluster with the answer given about whether, in the next ten years, your overall life will be better or worse seem to be concordant with the comments reported in [29]. The report contended that “as the effects of climate change become more widespread and significant, communities least able to respond are bearing the largest burden. In the United States, communities disadvantaged by a legacy of racial segregation and environmental injustice struggle with disparate health outcomes, are vulnerable to the effects of climate change (e.g., severe flooding in low-lying areas and extreme heat in urban neighbourhoods), and lack of sufficient resources to recover from and rebuild for resilience against future events (p. 2)”.

The results about whether citizens see climate change or energy conservation as an extremely serious problem or not a problem at all also seem to be concordant with previous studies that have analysed individual attitudes and behaviour concerning the acceptance of technologies, environmental regulation, or pro-environmental action [62,63,64,65]. Gkargkavouzi et al. [63] found that environmental behaviour measure revealed six dimensions: civic actions, policy support, recycling, transportation choices, behaviours in a household setting and consumerism. The study concluded that environmental concerns were more important drivers in explaining environmental regulation support and transportation choices.

It is not easy to find evidence of why American residents answering in Spanish are less likely to be in the unconvinced cluster. Based on the current lack of research specifically addressing the AP-ERE object, it can be speculated that sub-national culture may play a significant role. Jones et al. [66] discovered that Black Protestants and Hispanic Catholics were significantly more likely to anticipate that climate change would negatively affect them and others like them compared to White Protestants and White Catholics. Furthermore, Elgaaied-Gambier [67] discovered that the degree of support for environmental regulation is influenced by national culture. Using Hofstede’s model, the author identified specific cultural characteristics in European countries linked to support for lenient or stringent environmental regulations. This led to the conclusion that environmental regulations should be customised to fit the cultural traits of individual nations.

Young generations seem more unconvinced than other age segments regarding the effects of environmental regulation. The unconvinced state might be the fruit of the seeking information behaviour. In this respect, Whitmarsh et al. [36] found that “climate anxiety was higher amongst younger age groups, those with higher climate concern, higher generalised anxiety, lower mindfulness, higher nature relatedness, and more climate change information seeking behaviour. In addition, climate anxiety predicted some (but not all) types of pro-environmental action (p.1)”.

Regarding religion and religiosity, the results suggest that non-religious respondents and certain religious-affiliation categories exhibit higher unconvinced membership weights. In particular, Hindu, Muslim and atheist respondents show relatively higher unconvinced weights than several other categories. These categories are mentioned together only because they display similar descriptive patterns in the ANOVA results; they are not aggregated in the estimation. Besides the extant literature on the relationship between religion and environmental-related behaviour, the conclusions are unclear, and the relationship between science, religion and tradition is frequently disputable [68]. The authors empirically analysed to what extent religions and religiosity shape environment-related perceptions and practices, finding that the extant research shows that the world’s religions often hinder but sometimes promote pro-environmental values and behaviours. Newman et al. [69] concluded that some religious believers are more and less supportive of environmental regulation, with Evangelical Protestants the least supportive and Jewish and black Protestant members the most supportive.

Very liberal citizens are more unconvinced than other more conservative ideological segments about the ERE. This result is partly found in Wullenkord et al. [70] where left-wing ideological values are consistently aligned with climate anxiety and pro-environmental positions. However, as Giles [60] contended, environmental regulation needs to work better for regulators at the state and federal levels, the regulated and the public. It is necessary to take a practical vision, work together, have a solution-oriented stance, and sidestep the ideological debates of past decades. Thus, a solid nonpartisan platform could be developed for the benefit of the millennials and Generation Z.

Interestingly, gender and income segmentation covariates do not affect the similarity degree to the unconvinced representative American citizen. These results are consistent with Whitmarsh et al. [36], who found inconclusive results regarding income and gender, concluding that climate change anxiety could transcend social status, class, or wealth, being felt equally among differing groups. However, the authors also recognise that income is not the only indicator of wealth or privilege and that future research is needed to analyse inter-class climate change anxiety.

### Limitations of the study

The study is not exempt from criticism and limitations that can serve as fruitful lines of future research. First, from a methodological perspective, the AP-ERE scale could include more items that differentiate between regulation policies according to their categorisation as command-and-control or softer measures. Second, it might also be helpful to include other latent variables in the analysis that examine other environmental regulation policies that were not assessed with the current AP-ERE scale, for example, collective or social action through ER activism, influencing others, such as in churches and religious services, lobbying city-hall members in power, and choosing energy alternatives, avoiding fossil fuels. Third, our results showed that religious and other socioeconomic and demographic variables significantly influenced the AP-ERE synthetic indicator. However, a more quantitative method for assessing the relative importance of each variable using the AP-ERE indicator remains an issue that warrants further analysis. Moreover, the study only analyses the U.S. case using a cross-sectional dataset. It would be interesting to see whether the results can be generalised to other parts of the world, and, more generally, longitudinal research is essential to show whether AP-ERE is changing over time.

## Conclusions

This research analysed Americans’ perception of the effect of Environmental Regulation Effects (AP-ERE) using a dataset from the Pew Research Centre [4]. The study complemented the extant literature on the acceptance of the environmental regulation using for the first time a fuzzy-clustering approach that analysed how religious and other socio-economic traits affect this perception. A significant contribution of the study resided in the analysis of whether the convinced and unconvinced profiling is affected by a group of interesting covariates that included religiosity and religious beliefs. The results provided several insights into how religion intersected with Americans’ perceptions of environmental regulation effects.

The study included eleven variables not jointly examined in previous AP-ERE studies. The results showed that the three items included in the latent variable construction were not equally evaluated by the whole sample of respondents, being Americans more certain that much higher prices for fuel and electricity will be the norm in the next thirty years. Interestingly, Americans present more heterogeneity regarding the answers to the gradual loss of individual freedom. Additionally, the study included three variables associated with religion to analyse the relationship of these variables with the AP-ERE position, namely the self-assessment of being a religious person, religion and religious service attendance. For all the variables, it was found that they played a determinant role in determining the AP-ERE position.
