# Supplementary material for: A fuzzy clustering approach to understanding religious and socioeconomic determinants of public perceptions of environmental regulation in the United States
Source: PLoS One. 2026 Aug 7;21(8):e0334868. doi: 10.1371/journal.pone.0334868 (PMC13450767; doi:10.1371/journal.pone.0334868)
Supplement: Supplementary file 1 [file pone.0334868.s002.docx]

**PONE-D-25-51401**

**Title: Examining the Interplay of Religion, Socioeconomic Factors, and Perceptions of Environmental Regulation in the United States.**

The authors acknowledge the editor and reviewers for their comments, which improved the paper's presentation. This document outlines our responses to the editor and reviewers’ comments. Our responses are italicized and indicated by a “🡪” sign.

# Editor

Dear Dr ,

Thank you for submitting your manuscript to PLOS ONE. After careful consideration, we feel that it has merit but does not fully meet PLOS ONE’s publication criteria as it currently stands. Therefore, we invite you to submit a revised version of the manuscript that addresses the points raised during the review process.

**ACADEMIC EDITOR:**

I kindly ask XX to thoroughly check the comments of both reviewers and carefully address them in their revised manuscript. In relation to religious and social effects on opinion behavior in the U.S., I suggest that the authors check the outcomes in doi.org/10.1142/S0129183124501900 and in doi.org/10.1371/journal.pone.0331959, if relevant to their context in regulations.

It is important that the authors are diligent in their revisions, taking into account the changes suggested by the reviewers in their resubmission.

Please submit your revised manuscript by Mar 21 2026 11:59PM. If you will need more time than this to complete your revisions, please reply to this message or contact the journal office at [plosone@plos.org](mailto:plosone@plos.org). When you're ready to submit your revision, log on to https://www.editorialmanager.com/pone/ and select the 'Submissions Needing Revision' folder to locate your manuscript file.

Please include the following items when submitting your revised manuscript:

- A letter that responds to each point raised by the academic editor and reviewer(s). You should upload this letter as a separate file labeled 'Response to Reviewers'.
- A marked-up copy of your manuscript that highlights changes made to the original version. You should upload this as a separate file labeled 'Revised Manuscript with Track Changes'.
- An unmarked version of your revised paper without tracked changes. You should upload this as a separate file labeled 'Manuscript'.

If you would like to make changes to your financial disclosure, please include your updated statement in your cover letter. Guidelines for resubmitting your figure files are available below the reviewer comments at the end of this letter.

If applicable, we recommend that you deposit your laboratory protocols in protocols.io to enhance the reproducibility of your results. Protocols.io assigns your protocol its own identifier (DOI) so that it can be cited independently in the future. For instructions see: [https://journals.plos.org/plosone/s/submission-guidelines#loc-laboratory-protocols](https://track.editorialmanager.com/CL0/https:%2F%2Fjournals.plos.org%2Fplosone%2Fs%2Fsubmission-guidelines%23loc-laboratory-protocols/1/010f019c2c1f9aa7-aeba8ba6-b7a1-46c0-83fb-ca8e85b5497b-000000/b3wcAHM1HLkSpXxZIBylWKkmCvVJvxNVSYh-pEYeUG4=249). Additionally, PLOS ONE offers an option for publishing peer-reviewed Lab Protocol articles, which describe protocols hosted on protocols.io. Read more information on sharing protocols at [https://plos.org/protocols?utm_medium=editorial-email&utm_source=authorletters&utm_campaign=protocols](https://track.editorialmanager.com/CL0/https:%2F%2Fplos.org%2Fprotocols%3Futm_medium=editorial-email%26utm_source=authorletters%26utm_campaign=protocols/1/010f019c2c1f9aa7-aeba8ba6-b7a1-46c0-83fb-ca8e85b5497b-000000/CtGo9OK_pnQGSn1wfdauYIuoj9v6K62zztPEathkTnw=249).

We look forward to receiving your revised manuscript.

Kind regards,

Omar El Deeb
Academic Editor
PLOS One

Journal Requirements:

When submitting your revision, we need you to address these additional requirements.

1. Please ensure that your manuscript meets PLOS ONE's style requirements, including those for file naming. The PLOS ONE style templates can be found at

<https://journals.plos.org/plosone/s/file?id=wjVg/PLOSOne_formatting_sample_main_body.pdf> and

[https://journals.plos.org/plosone/s/file?id=ba62/PLOSOne_formatting_sample_title_authors_affiliations.pdf](https://track.editorialmanager.com/CL0/https:%2F%2Fjournals.plos.org%2Fplosone%2Fs%2Ffile%3Fid=ba62%2FPLOSOne_formatting_sample_title_authors_affiliations.pdf/1/010f019c2c1f9aa7-aeba8ba6-b7a1-46c0-83fb-ca8e85b5497b-000000/f_bUkjkERemd5WyaSTZueCqlJw2yB0i2mfGWG8NCF1M=249)

🡪 We thank the Editor for this clarification. We have carefully revised the manuscript to ensure full compliance with PLOS ONE’s formatting and style requirements. Specifically, we have adapted the structure, headings, references, and file naming conventions according to the official templates provided.

2. We noticed you have some minor occurrence of overlapping text with the following previous publication(s), which needs to be addressed:

- <https://doi.org/10.1016/j.ssaho.2025.101529>

In your revision ensure you cite all your sources (including your own works), and quote or rephrase any duplicated text outside the methods section. Further consideration is dependent on these concerns being addressed.

🡪 We thank the Editor for raising this important issue. We have carefully reviewed the manuscript to identify and address any overlapping text with our previous publication.

3. Thank you for stating the following financial disclosure: [The author(s) disclosed receipt of the following financial support for the research, authorship, and/or publication of this article: This study was funded by the research fellowship “Catalina Ruiz,” provided by Gobierno de Canarias and the Agencia Canaria De Investigación Innovación Y Sociedad De La Información (ACIISI) and European Social Fund, through the Universidad de Las Palmas de Gran Canaria (Spain).].

Please state what role the funders took in the study.  If the funders had no role, please state: ""The funders had no role in study design, data collection and analysis, decision to publish, or preparation of the manuscript.""

If this statement is not correct you must amend it as needed.

Please include this amended Role of Funder statement in your cover letter; we will change the online submission form on your behalf.

🡪 We confirm that the funders had no role in the study design, data collection and analysis, decision to publish, or preparation of the manuscript. This statement has been included in the revised cover letter as requested, and we kindly ask the editorial office to update the submission system accordingly.

4. We note that the grant information you provided in the ‘Funding Information’ and ‘Financial Disclosure’ sections do not match.

When you resubmit, please ensure that you provide the correct grant numbers for the awards you received for your study in the ‘Funding Information’ section.

🡪 We have carefully reviewed and corrected the funding information to ensure full consistency between the ‘Funding Information’ and ‘Financial Disclosure’ sections. The revised manuscript now includes the correct grant details and funding information across all sections.

5. Thank you for uploading your study's underlying data set. Unfortunately, the repository you have noted in your Data Availability statement does not qualify as an acceptable data repository according to PLOS's standards.

🡪 We thank the Editor for highlighting this issue. We have updated the Data Availability Statement to include the corresponding DOI and access link to the repository. This ensures transparency, reproducibility, and full compliance with PLOS data requirements.

At this time, please upload the minimal data set necessary to replicate your study's findings to a stable, public repository (such as figshare or Dryad) and provide us with the relevant URLs, DOIs, or accession numbers that may be used to access these data. For a list of recommended repositories and additional information on PLOS standards for data deposition, please see [https://journals.plos.org/plosone/s/recommended-repositories](https://track.editorialmanager.com/CL0/https:%2F%2Fjournals.plos.org%2Fplosone%2Fs%2Frecommended-repositories/1/010f019c2c1f9aa7-aeba8ba6-b7a1-46c0-83fb-ca8e85b5497b-000000/uOellFMdJ7vgd20s6AokzbRi_m9feg53Yc2NQjZbDr8=249).

6. Please include captions for your Supporting Information files at the end of your manuscript, and update any in-text citations to match accordingly. Please see our Supporting Information guidelines for more information: <http://journals.plos.org/plosone/s/supporting-information>.

7. If the reviewer comments include a recommendation to cite specific previously published works, please review and evaluate these publications to determine whether they are relevant and should be cited. There is no requirement to cite these works unless the editor has indicated otherwise. 

[Note: HTML markup is below. Please do not edit.]

- See below how we have responded to each of the comments made by the referees.

# Reviewers' comments

Reviewer's Responses to Questions

**Comments to the Author**

1. Is the manuscript technically sound, and do the data support the conclusions?

The manuscript must describe a technically sound piece of scientific research with data that supports the conclusions. Experiments must have been conducted rigorously, with appropriate controls, replication, and sample sizes. The conclusions must be drawn appropriately based on the data presented.

Reviewer #1: Yes

Reviewer #2: No

2. Has the statistical analysis been performed appropriately and rigorously?

Reviewer #1: Yes

Reviewer #2: I Don't Know

3. Have the authors made all data underlying the findings in their manuscript fully available?

The [PLOS Data policy](http://track.editorialmanager.com/CL0/http:%2F%2Fwww.plosone.org%2Fstatic%2Fpolicies.action%23sharing/1/010f019c2c1f9aa7-aeba8ba6-b7a1-46c0-83fb-ca8e85b5497b-000000/iUb0yRdcQh3PCQBGumHahu2wXQ0gqmD7SdUAZtKXJNI=249) requires authors to make all data underlying the findings described in their manuscript fully available without restriction, with rare exception (please refer to the Data Availability Statement in the manuscript PDF file). The data should be provided as part of the manuscript or its supporting information, or deposited to a public repository. For example, in addition to summary statistics, the data points behind means, medians and variance measures should be available. If there are restrictions on publicly sharing data—e.g. participant privacy or use of data from a third party—those must be specified.

Reviewer #1: Yes

Reviewer #2: Yes

4. Is the manuscript presented in an intelligible fashion and written in standard English?

PLOS ONE does not copyedit accepted manuscripts, so the language in submitted articles must be clear, correct, and unambiguous. Any typographical or grammatical errors should be corrected at revision, so please note any specific errors here.

Reviewer #1: Yes

Reviewer #2: Yes

5. Review Comments to the Author

Please use the space provided to explain your answers to the questions above. You may also include additional comments for the author, including concerns about dual publication, research ethics, or publication ethics. (Please upload your review as an attachment if it exceeds 20,000 characters)

Reviewer #1: Generally this is a well-written manuscript. You have answered the study question. The statistical analyses were adequate and your references were mainly up-to-date. Perhaps you can discuss about the limitations to the study before making a concluding remark.

🡪 We thank the reviewer for the positive and constructive feedback. In response to this suggestion, we have expanded the limitations of the study to provide a more balanced interpretation of the findings. Specifically, we have added a dedicated paragraph in the “Limitations of the study” section (after Discussions), where we discuss methodological constraints, including the use of a three-item proxy for perceived environmental regulation effects, the cross-sectional nature of the dataset, and the potential lack of generalisability beyond the U.S. context.

Reviewer #2: Some of the initial premises are unclear and appear to present an ideological stance and assumptions. Why is environmental regulation adequately represented by the proxy questions: (1) Much higher prices for fuel and electricity; (2) Gradual loss of individual freedoms; and (3) fewer jobs and declining pay in industries that depend on fossil fuels? These seem like issues which could / should be much more widely interpreted than environmental regulation. The author’s definition of Regulation needs to be given upfront.

🡪 We thank the reviewer for this important and insightful comment. We acknowledge that the initial version of the manuscript did not sufficiently clarify the conceptualisation of environmental regulation within the scope of this study.

To address this, we have revised the Introduction to provide a clearer, more explicit definition of environmental regulation as a broad set of policies and institutional interventions that aim to mitigate environmental externalities. Importantly, we now emphasise that the study does not measure environmental regulation per se, but rather individuals’ perceptions of its potential socio-economic consequences.

Additionally, we have clarified that the three proxy items (energy prices, individual freedoms, and employment in fossil-fuel sectors) are derived from the Pew Research Centre survey and are interpreted as perceived outcomes associated with environmental regulation, rather than an exhaustive representation of the concept. This distinction is now explicitly stated to avoid any potential misinterpretation.

This continues throughout the paper. For example: “The environmental regulation to reduce greenhouse gas emissions will lead to higher electricity prices (Lee, 2022).” Firstly, higher electricity prices to who? Higher costs in generation are not necessarily transferred directly to the customer, that is a choice. Secondly, the seminal work on environmental regulation highlights that well designed regulation acts to reduce costs, and is designed to drive compliance, please see Porter, M. E., & Linde, C. V. D. (1995). Toward a new conception of the environment-competitiveness relationship. Journal of economic perspectives, 9(4), 97-118. There are multiple assessments of the application of the ‘Porter Hypothesis’ but this gets only a tangential mention in the paper and is not explained.

🡪 We agree that the relationship between environmental regulation and electricity prices is complex and context-dependent. In response, we have revised the Literature Review to clarify that increases in electricity prices are not automatic or universal outcomes of environmental regulation, but rather depend on market structures, policy design, and regulatory choices. We now explicitly acknowledge that cost pass-through to consumers is not guaranteed.

Furthermore, we have expanded the discussion of the Porter Hypothesis by incorporating a more detailed explanation of how well-designed environmental regulation can foster innovation, improve efficiency, and potentially reduce long-term costs. This theoretical perspective is now more clearly integrated into the manuscript, providing a more balanced and nuanced interpretation of the economic impacts of environmental regulation.

There are some very strong statements made with very limited justification. For example: “Environmental regulations have caused an important decline in the coal industry worldwide, leading to job losses in various industry sectors, including mining, transportation, and power generation.” I don’t think you can attribute the shifts in the coal industry to environmental regulation alone. In fact, a very quick check highlights that natural gas may have as significant an impact as environmental regulation, for example: Houser, T., Bordoff, J., & Marsters, P. (2017). Can coal make a comeback?. New York, NY: Columbia Center on Global Energy Policy.

🡪 We agree that attributing the decline of the coal industry solely to environmental regulation is an oversimplification. We now explicitly acknowledge that the decline of the coal industry is driven by multiple factors, including technological change, market dynamics, and, in particular, the increasing competitiveness of natural gas, as highlighted by Houser et al. (2017).

The authors say that “We could not find any scale developed to measure the environmental regulation effects perception”. There is a well-established literature base on the perception of regulation. See Valerie Braithwaite’s work, for example: Braithwaite, V. (2003). Dancing with tax authorities: Motivational postures and non-compliant actions. Taxing democracy, 3, 15-39. Or Braithwaite, V. (2017). Closing the gap between regulation and the community. Regulatory theory: Foundations and applications, 59(2), 25-42. It isn’t clear why the authors would not use these as a starting point. In addition, the perceived and actual effects of environmental regulation, for example on competitiveness, cost of implementation, etc. Have been well documented and discussed across multiple papers, to pick out one example: López-Gamero, M. D., Molina-Azorín, J. F., & Claver-Cortés, E. (2010). The potential of environmental regulation to change managerial perception, environmental management, competitiveness and financial performance. Journal of Cleaner Production, 18(10-11), 963-974.

🡪We thank the reviewer for pointing out this relevant body of literature. In response, we have expanded the Literature Review to incorporate key references, including Braithwaite (2017), and to clarify the distinction between general regulatory perception (e.g., compliance attitudes and motivational postures) and the specific focus of our study.

We now explicitly state that, while there is an established literature on perceptions of regulation, there is no widely accepted scale specifically designed to measure perceived socio-economic effects of environmental regulation as operationalised in this study. Additionally, we have incorporated relevant contributions, such as those of López-Gamero et al. (2010), to strengthen the paper's theoretical grounding.

There is a huge dataset generated through the survey aiming “to explore the relationship between American's religious beliefs and their views on climate change and the environment”. This looks like a fascinating dataset which could be used very usefully to understand the perceptions of different groups within the USA. However, it seems erroneous to frame this as a paper about environmental regulation. I would recommend the authors review and rethink their use and presentation of the data.

🡪We thank the reviewer for this thoughtful comment. We agree that the dataset provides a rich opportunity to explore broader attitudes towards climate change and the environment. However, the specific contribution of this study lies in analysing how individuals perceive the potential socio-economic effects of environmental regulation, using the AP-ERE indicator derived from the Pew survey items. To address the reviewer’s concern, we have clarified the scope and positioning of the paper throughout the manuscript. In particular, we have revised the Introduction and Discussion sections to emphasise that the study focuses on perceived consequences of environmental regulation, rather than on environmental attitudes in general.

6. PLOS authors have the option to publish the peer review history of their article ([what does this mean?](https://track.editorialmanager.com/CL0/https:%2F%2Fjournals.plos.org%2Fplosone%2Fs%2Feditorial-and-peer-review-process%23loc-peer-review-history/1/010f019c2c1f9aa7-aeba8ba6-b7a1-46c0-83fb-ca8e85b5497b-000000/zvS9bktHA3BtFL9UjKLQayLBsJ2Oz6_C7WXqouYGBeQ=249)). If published, this will include your full peer review and any attached files.


If you choose “no”, your identity will remain anonymous but your review may still be made public.


**Do you want your identity to be public for this peer review?** For information about this choice, including consent withdrawal, please see our [Privacy Policy](https://track.editorialmanager.com/CL0/https:%2F%2Fwww.plos.org%2Fprivacy-policy/1/010f019c2c1f9aa7-aeba8ba6-b7a1-46c0-83fb-ca8e85b5497b-000000/yxR3ltWhn-L3izu8hL8qcvofqjMhTKwF0RekqRyBpFk=249).

Reviewer #1: **Yes:** Dr Ahmad Farouk Musa

Reviewer #2: **Yes:** Claire Grisaffi

🡪We would like to sincerely thank the Academic Editor and both reviewers for their valuable comments and constructive suggestions. Their feedback has been extremely helpful in improving the clarity, rigour, and overall quality of our manuscript. We believe that the revisions made in response to their insights have significantly strengthened the paper.
