# Supplementary material for: A fuzzy clustering approach to understanding religious and socioeconomic determinants of public perceptions of environmental regulation in the United States
Source: PLoS One. 2026 Aug 7;21(8):e0334868. doi: 10.1371/journal.pone.0334868 (PMC13450767; doi:10.1371/journal.pone.0334868)
Supplement: Supplementary file 2 [file pone.0334868.s003.docx]

**PONE-D-25-51401R1**

A Fuzzy Clustering Approach to Understanding Religious and Socioeconomic Determinants of Public Perceptions of Environmental Regulation in the United States

PLOS One

The authors acknowledge the editor and reviewers for their comments, which improved the paper's presentation. This document outlines our responses to the editor and reviewers’ comments. Our responses are italicized and indicated by a “🡪” sign.

Dear Dr.  ,

Thank you for submitting your manuscript to PLOS ONE. After careful consideration, we feel that it has merit but does not fully meet PLOS ONE’s publication criteria as it currently stands. Therefore, we invite you to submit a revised version of the manuscript that addresses the points raised during the review process.

Please submit your revised manuscript by Jun 05 2026 11:59PM. If you will need more time than this to complete your revisions, please reply to this message or contact the journal office at [plosone@plos.org](mailto:plosone@plos.org). When you're ready to submit your revision, log on to https://www.editorialmanager.com/pone/ and select the 'Submissions Needing Revision' folder to locate your manuscript file.

Please include the following items when submitting your revised manuscript:

- A letter that responds to each point raised by the academic editor and reviewer(s). You should upload this letter as a separate file labeled 'Response to Reviewers'.
- A marked-up copy of your manuscript that highlights changes made to the original version. You should upload this as a separate file labeled 'Revised Manuscript with Track Changes'.
- An unmarked version of your revised paper without tracked changes. You should upload this as a separate file labeled 'Manuscript'.

If you would like to make changes to your financial disclosure, please include your updated statement in your cover letter. Guidelines for resubmitting your figure files are available below the reviewer comments at the end of this letter.

If applicable, we recommend that you deposit your laboratory protocols in protocols.io to enhance the reproducibility of your results. Protocols.io assigns your protocol its own identifier (DOI) so that it can be cited independently in the future. For instructions see: <https://journals.plos.org/plosone/s/submission-guidelines#loc-laboratory-protocols>.

Additionally, PLOS ONE offers an option for publishing peer-reviewed Lab Protocol articles, which describe protocols hosted on protocols.io. Read more information on sharing protocols at <https://plos.org/protocols?utm_medium=editorial-email&utm_source=authorletters&utm_campaign=protocols>.

As the corresponding author, your ORCID iD is verified in the submission system and will appear in the published article. PLOS supports the use of ORCID, and we encourage all coauthors to register for an ORCID iD and use it as well. Please encourage your coauthors to verify their ORCID iD within the submission system before final acceptance, as unverified ORCID iDs will not appear in the published article. *Only* the individual author can complete the verification step; PLOS staff *cannot* verify ORCID iDs on behalf of authors.

We look forward to receiving your revised manuscript.

Kind regards,

Omar El Deeb

Academic Editor

PLOS One

Journal Requirements:

1. If the reviewer comments include a recommendation to cite specific previously published works, please review and evaluate these publications to determine whether they are relevant and should be cited. There is no requirement to cite these works unless the editor has indicated otherwise.

🡪We thank the Academic Editor for this suggestion. We have carefully considered the reviewer’s comments and the literature. Where relevant, we have added references that support the theoretical framing of the revised manuscript.

2. Please review your reference list to ensure that it is complete and correct. If you have cited papers that have been retracted, please include the rationale for doing so in the manuscript text, or remove these references and replace them with relevant current references. Any changes to the reference list should be mentioned in the rebuttal letter that accompanies your revised manuscript. If you need to cite a retracted article, indicate the article’s retracted status in the References list and also include a citation and full reference for the retraction notice.

🡪The reference list has been checked thoroughly to ensure all entries are complete, correct and comply with the journal’s requirements. We also checked the cited literature for retraction notices and found no retracted articles among the references used in the revised manuscript.

[Note: HTML markup is below. Please do not edit.]

Reviewer's Responses to Questions

Comments to the Author

1. If the authors have adequately addressed your comments raised in a previous round of review and you feel that this manuscript is now acceptable for publication, you may indicate that here to bypass the “Comments to the Author” section, enter your conflict of interest statement in the “Confidential to Editor” section, and submit your "Accept" recommendation.

Reviewer #3: (No Response)

2. Is the manuscript technically sound, and do the data support the conclusions?

The manuscript must describe a technically sound piece of scientific research with data that supports the conclusions. Experiments must have been conducted rigorously, with appropriate controls, replication, and sample sizes. The conclusions must be drawn appropriately based on the data presented.

Reviewer #3: Yes

3. Has the statistical analysis been performed appropriately and rigorously?

Reviewer #3: Yes

4. Have the authors made all data underlying the findings in their manuscript fully available?

The [PLOS Data policy](http://www.plosone.org/static/policies.action#sharing) requires authors to make all data underlying the findings described in their manuscript fully available without restriction, with rare exception (please refer to the Data Availability Statement in the manuscript PDF file). The data should be provided as part of the manuscript or its supporting information, or deposited to a public repository. For example, in addition to summary statistics, the data points behind means, medians and variance measures should be available. If there are restrictions on publicly sharing data—e.g. participant privacy or use of data from a third party—those must be specified.

Reviewer #3: No

5. Is the manuscript presented in an intelligible fashion and written in standard English?

PLOS ONE does not copyedit accepted manuscripts, so the language in submitted articles must be clear, correct, and unambiguous. Any typographical or grammatical errors should be corrected at revision, so please note any specific errors here.

Reviewer #3: Yes

6. Review Comments to the Author

Please use the space provided to explain your answers to the questions above. You may also include additional comments for the author, including concerns about dual publication, research ethics, or publication ethics. (Please upload your review as an attachment if it exceeds 20,000 characters)

Reviewer #3: A Fuzzy Clustering Approach to Understanding Religious and Socioeconomic Determinants of Public Perceptions of Environmental Regulation in the United States

Reviewer Report:

1. The manuscript identifies three perceived outcomes of environmental regulation:
(i) higher fuel and electricity prices: This outcome can plausibly be linked to environmental regulation through mechanisms such as carbon pricing, emissions standards, and compliance costs imposed on energy producers. However, price is determined by the general supply-demand Dynamics.

🡪We thank the reviewer for this useful comment. We have clarified in the Literature Review (Environmental Regulation Impacts) that higher fuel and electricity prices are a perceived consequence of environmental regulation rather than an objective price effect. We now also acknowledge that energy prices are shaped by supply-demand dynamics, market volatility, infrastructure constraints, geopolitical shocks and policy design.

(ii) loss of individual freedoms: This is not inherently a direct consequence of environmental regulation but rather reflects public perception of regulatory overreach. The authors should clarify that this dimension captures subjective interpretations (e.g., restrictions on driving a 4000 cc) rather than objective regulatory outcomes.

🡪We thank the reviewer for this important clarification. We have revised the Literature Review (Environmental Regulation Effects Surveys). Now, it is specified that this item captures respondents’ subjective perception of regulatory overreach, rather than a loss of individual freedoms that could be objectively observed.

(iii) reduced employment in fossil-fuel-based industries: While job losses may occur in carbon-intensive sectors due to environmental policies, attributing them solely to regulation is overly simplistic. Structural changes such as automation and market transitions toward renewable energy also play a role.

🡪We agree with the reviewer. As we have reviewed in the Literature Review (Environmental Regulation Impacts section), the decline in employment in fossil-fuel-dependent industries cannot be attributed solely to environmental regulation. The revised manuscript now recognises the roles of automation, market restructuring, and the transition to renewable energy.

Without clarifications, the constructs appear theoretically weak. It should be strictly stressed that these are perceptions, thoughts of US consumers. The authors should reframe these variables explicitly as perceived consequences rather than objective effects. The theoretical linkage between these outcomes and environmental regulation is currently underdeveloped and, in parts, conceptually inconsistent. (Still)

🡪We thank the reviewer for this valuable observation. We have revised the Abstract, Introduction, Variables section, Methodology, Results, and Discussions to clarify that the three items are indicators of perception. The AP-ERE index is explicitly described as a measure of respondents' perceived likelihood of potential consequences, rather than as evidence of objective or causal regulatory effects.

2. Typographical Error

The phrase “Mormon (Church of Jesus Christ” “)” contains a formatting error (missing closing parenthesis or incorrect punctuation).

🡪We thank the reviewer for pointing this out. We have corrected the formatting error in Tables 2 and 3 and now report the category consistently as “Mormon/Latter-day Saint” throughout the manuscript.

3. Inconsistency in Grouping Religious Categories

There is a lack of consistency between the table presentation and the textual interpretation:
In Table 2-3, religious groups such as Mormons and Protestants are presented as distinct categories, yet they are combined in the text.

Similarly, Atheists, Muslims, and Hindus are grouped together in the text despite being separate categories in the table.

Provide a clear rationale for aggregating categories (e.g., theoretical similarity, cluster analysis results, or sample size limitations). Ensure consistency between tables and narrative.
If grouping is necessary, consider reporting both disaggregated and aggregated results for transparency.

🡪We thank the reviewer for this constructive comment. As we explained in the Variables section, religious categories were analysed separately rather than formally aggregated. We also revised the Results and Discussion sections to align with Tables 2 and 3. Some categories are mentioned together for interpretative purposes only, as they exhibit similar descriptive patterns.

7. PLOS authors have the option to publish the peer review history of their article ([what does this mean?](https://journals.plos.org/plosone/s/editorial-and-peer-review-process#loc-peer-review-history)). If published, this will include your full peer review and any attached files.

If you choose “no”, your identity will remain anonymous but your review may still be made public.

Do you want your identity to be public for this peer review? For information about this choice, including consent withdrawal, please see our [Privacy Policy](https://www.plos.org/privacy-policy).

Reviewer #3: No

[NOTE: If reviewer comments were submitted as an attachment file, they will be attached to this email and accessible via the submission site. Please log into your account, locate the manuscript record, and check for the action link "View Attachments". If this link does not appear, there are no attachment files.]

To ensure your figures meet our technical requirements, please review our figure guidelines: https://journals.plos.org/plosone/s/figures

You may also use PLOS’s free figure tool, NAAS, to help you prepare publication quality figures: https://journals.plos.org/plosone/s/figures#loc-tools-for-figure-preparation.

NAAS will assess whether your figures meet our technical requirements by comparing each figure against our figure specifications.

We are grateful to the Academic Editor and Reviewers for their careful reading of the manuscript and constructive comments. Their comments were useful in clarifying the study's conceptual framing, strengthening the interpretation of the perception-based indicators, and improving the consistency of the presentation. We think that the revised manuscript is now clearer, more precise and better adapted to the aims and standards of PLOS ONE.
