# Supplementary material for: A fuzzy clustering approach to understanding religious and socioeconomic determinants of public perceptions of environmental regulation in the United States
Source: PLoS One. 2026 Aug 7;21(8):e0334868. doi: 10.1371/journal.pone.0334868 (PMC13450767; doi:10.1371/journal.pone.0334868)
Supplement: Peer review history

# Peer review: Decision Letter 0

**DOI:** 10.1371/journal.pone.0334868.r001

**Authors:** Omar El Deeb

4 Feb 2026

PONE-D-25-51401

Examining the Interplay of Religion, Socioeconomic Factors, and Perceptions of Environmental Regulation in the United States.

PLOS One

Dear Dr. Indelicato,

Thank you for submitting your manuscript to PLOS ONE. After careful consideration, we feel that it has merit but does not fully meet PLOS ONE’s publication criteria as it currently stands. Therefore, we invite you to submit a revised version of the manuscript that addresses the points raised during the review process.

**ACADEMIC EDITOR:**

I kindly ask Dr Indelicato to thoroughly check the comments of both reviewers and carefully address them in their revised manuscript. In relation to religious and social effects on opinion behavior in the U.S., I suggest that the authors check the outcomes in doi.org/10.1142/S0129183124501900 and in doi.org/10.1371/journal.pone.0331959, if relevant to their context in regulations.

It is important that the authors are diligent in their revisions, taking into account the changes suggested by the reviewers in their resubmission.

Please submit your revised manuscript by Mar 21 2026 11:59PM. If you will need more time than this to complete your revisions, please reply to this message or contact the journal office at plosone@plos.org. When you're ready to submit your revision, log on to https://www.editorialmanager.com/pone/ and select the 'Submissions Needing Revision' folder to locate your manuscript file.

Please include the following items when submitting your revised manuscript:

- A letter that responds to each point raised by the academic editor and reviewer(s). You should upload this letter as a separate file labeled 'Response to Reviewers'.
- A marked-up copy of your manuscript that highlights changes made to the original version. You should upload this as a separate file labeled 'Revised Manuscript with Track Changes'.
- An unmarked version of your revised paper without tracked changes. You should upload this as a separate file labeled 'Manuscript'.

If you would like to make changes to your financial disclosure, please include your updated statement in your cover letter. Guidelines for resubmitting your figure files are available below the reviewer comments at the end of this letter.

If applicable, we recommend that you deposit your laboratory protocols in protocols.io to enhance the reproducibility of your results. Protocols.io assigns your protocol its own identifier (DOI) so that it can be cited independently in the future. For instructions see: https://journals.plos.org/plosone/s/submission-guidelines#loc-laboratory-protocols. Additionally, PLOS ONE offers an option for publishing peer-reviewed Lab Protocol articles, which describe protocols hosted on protocols.io. Read more information on sharing protocols at https://plos.org/protocols?utm_medium=editorial-email&utm_source=authorletters&utm_campaign=protocols.

We look forward to receiving your revised manuscript.

Kind regards,

Omar El Deeb

Academic Editor

PLOS One

Journal Requirements:

When submitting your revision, we need you to address these additional requirements.

1. Please ensure that your manuscript meets PLOS ONE's style requirements, including those for file naming. The PLOS ONE style templates can be found at

https://journals.plos.org/plosone/s/file?id=wjVg/PLOSOne_formatting_sample_main_body.pdf and

https://journals.plos.org/plosone/s/file?id=ba62/PLOSOne_formatting_sample_title_authors_affiliations.pdf

2. We noticed you have some minor occurrence of overlapping text with the following previous publication(s), which needs to be addressed:

- https://doi.org/10.1016/j.ssaho.2025.101529

In your revision ensure you cite all your sources (including your own works), and quote or rephrase any duplicated text outside the methods section. Further consideration is dependent on these concerns being addressed.

3. Thank you for stating the following financial disclosure: [The author(s) disclosed receipt of the following financial support for the research, authorship, and/or publication of this article: This study was funded by the research fellowship “Catalina Ruiz,” provided by Gobierno de Canarias and the Agencia Canaria De Investigación Innovación Y Sociedad De La Información (ACIISI) and European Social Fund, through the Universidad de Las Palmas de Gran Canaria (Spain).].

Please state what role the funders took in the study.  If the funders had no role, please state: ""The funders had no role in study design, data collection and analysis, decision to publish, or preparation of the manuscript.""

If this statement is not correct you must amend it as needed.

Please include this amended Role of Funder statement in your cover letter; we will change the online submission form on your behalf.

4. We note that the grant information you provided in the ‘Funding Information’ and ‘Financial Disclosure’ sections do not match.

When you resubmit, please ensure that you provide the correct grant numbers for the awards you received for your study in the ‘Funding Information’ section.

5. Thank you for uploading your study's underlying data set. Unfortunately, the repository you have noted in your Data Availability statement does not qualify as an acceptable data repository according to PLOS's standards.

At this time, please upload the minimal data set necessary to replicate your study's findings to a stable, public repository (such as figshare or Dryad) and provide us with the relevant URLs, DOIs, or accession numbers that may be used to access these data. For a list of recommended repositories and additional information on PLOS standards for data deposition, please see https://journals.plos.org/plosone/s/recommended-repositories.

6. Please include captions for your Supporting Information files at the end of your manuscript, and update any in-text citations to match accordingly. Please see our Supporting Information guidelines for more information: http://journals.plos.org/plosone/s/supporting-information.

7. If the reviewer comments include a recommendation to cite specific previously published works, please review and evaluate these publications to determine whether they are relevant and should be cited. There is no requirement to cite these works unless the editor has indicated otherwise.

[Note: HTML markup is below. Please do not edit.]

Reviewers' comments:

Reviewer's Responses to Questions

**Comments to the Author**

1. Is the manuscript technically sound, and do the data support the conclusions?

The manuscript must describe a technically sound piece of scientific research with data that supports the conclusions. Experiments must have been conducted rigorously, with appropriate controls, replication, and sample sizes. The conclusions must be drawn appropriately based on the data presented.

Reviewer #1: Yes

Reviewer #2: No

**********

2. Has the statistical analysis been performed appropriately and rigorously?

Reviewer #1: Yes

Reviewer #2: I Don't Know

**********

3. Have the authors made all data underlying the findings in their manuscript fully available?

The PLOS Data policy requires authors to make all data underlying the findings described in their manuscript fully available without restriction, with rare exception (please refer to the Data Availability Statement in the manuscript PDF file). The data should be provided as part of the manuscript or its supporting information, or deposited to a public repository. For example, in addition to summary statistics, the data points behind means, medians and variance measures should be available. If there are restrictions on publicly sharing data—e.g. participant privacy or use of data from a third party—those must be specified.

Reviewer #1: Yes

Reviewer #2: Yes

**********

4. Is the manuscript presented in an intelligible fashion and written in standard English?

PLOS ONE does not copyedit accepted manuscripts, so the language in submitted articles must be clear, correct, and unambiguous. Any typographical or grammatical errors should be corrected at revision, so please note any specific errors here.

Reviewer #1: Yes

Reviewer #2: Yes

**********

5. Review Comments to the Author

Please use the space provided to explain your answers to the questions above. You may also include additional comments for the author, including concerns about dual publication, research ethics, or publication ethics. (Please upload your review as an attachment if it exceeds 20,000 characters)

Reviewer #1: Generally this is a well-written manuscript. You have answered the study question. The statistical analyses were adequate and your references were mainly up-to-date. Perhaps you can discuss about the limitations to the study before making a concluding remark.

Reviewer #2: Some of the initial premises are unclear and appear to present an ideological stance and assumptions. Why is environmental regulation adequately represented by the proxy questions: (1) Much higher prices for fuel and electricity; (2) Gradual loss of individual freedoms; and (3) fewer jobs and declining pay in industries that depend on fossil fuels? These seem like issues which could / should be much more widely interpreted than environmental regulation. The author’s definition of Regulation needs to be given upfront.

This continues throughout the paper. For example: “The environmental regulation to reduce greenhouse gas emissions will lead to higher electricity prices (Lee, 2022).” Firstly, higher electricity prices to who? Higher costs in generation are not necessarily transferred directly to the customer, that is a choice. Secondly, the seminal work on environmental regulation highlights that well designed regulation acts to reduce costs, and is designed to drive compliance, please see Porter, M. E., & Linde, C. V. D. (1995). Toward a new conception of the environment-competitiveness relationship. Journal of economic perspectives, 9(4), 97-118. There are multiple assessments of the application of the ‘Porter Hypothesis’ but this gets only a tangential mention in the paper and is not explained.

There are some very strong statements made with very limited justification. For example: “Environmental regulations have caused an important decline in the coal industry worldwide, leading to job losses in various industry sectors, including mining, transportation, and power generation.” I don’t think you can attribute the shifts in the coal industry to environmental regulation alone. In fact, a very quick check highlights that natural gas may have as significant an impact as environmental regulation, for example: Houser, T., Bordoff, J., & Marsters, P. (2017). Can coal make a comeback?. New York, NY: Columbia Center on Global Energy Policy.

The authors say that “We could not find any scale developed to measure the environmental regulation effects perception”. There is a well-established literature base on the perception of regulation. See Valerie Braithwaite’s work, for example: Braithwaite, V. (2003). Dancing with tax authorities: Motivational postures and non-compliant actions. Taxing democracy, 3, 15-39. Or Braithwaite, V. (2017). Closing the gap between regulation and the community. Regulatory theory: Foundations and applications, 59(2), 25-42. It isn’t clear why the authors would not use these as a starting point. In addition, the perceived and actual effects of environmental regulation, for example on competitiveness, cost of implementation, etc. Have been well documented and discussed across multiple papers, to pick out one example: López-Gamero, M. D., Molina-Azorín, J. F., & Claver-Cortés, E. (2010). The potential of environmental regulation to change managerial perception, environmental management, competitiveness and financial performance. Journal of Cleaner Production, 18(10-11), 963-974.

There is a huge dataset generated through the survey aiming “to explore the relationship between American's religious beliefs and their views on climate change and the environment”. This looks like a fascinating dataset which could be used very usefully to understand the perceptions of different groups within the USA. However, it seems erroneous to frame this as a paper about environmental regulation. I would recommend the authors review and rethink their use and presentation of the data.

**********

6. PLOS authors have the option to publish the peer review history of their article (what does this mean?). If published, this will include your full peer review and any attached files.

If you choose “no”, your identity will remain anonymous but your review may still be made public.

**Do you want your identity to be public for this peer review?**  For information about this choice, including consent withdrawal, please see our Privacy Policy.

Reviewer #1: **Yes:** Dr Ahmad Farouk Musa

Reviewer #2: **Yes:** Claire Grisaffi

**********

[NOTE: If reviewer comments were submitted as an attachment file, they will be attached to this email and accessible via the submission site. Please log into your account, locate the manuscript record, and check for the action link "View Attachments". If this link does not appear, there are no attachment files.]

To ensure your figures meet our technical requirements, please review our figure guidelines: https://journals.plos.org/plosone/s/figures

You may also use PLOS’s free figure tool, NAAS, to help you prepare publication quality figures: https://journals.plos.org/plosone/s/figures#loc-tools-for-figure-preparation.

NAAS will assess whether your figures meet our technical requirements by comparing each figure against our figure specifications.

# Author comment: Author response to Decision Letter 1

**DOI:** 10.1371/journal.pone.0334868.r002

1 Apr 2026

PONE-D-25-51401

Title: Examining the Interplay of Religion, Socioeconomic Factors, and Perceptions of Environmental Regulation in the United States.

The authors acknowledge the editor and reviewers for their comments, which improved the paper's presentation. This document outlines our responses to the editor and reviewers’ comments. Our responses are italicized and indicated by a “�” sign.

Editor

Dear Dr ,

Thank you for submitting your manuscript to PLOS ONE. After careful consideration, we feel that it has merit but does not fully meet PLOS ONE’s publication criteria as it currently stands. Therefore, we invite you to submit a revised version of the manuscript that addresses the points raised during the review process.

ACADEMIC EDITOR:

I kindly ask XX to thoroughly check the comments of both reviewers and carefully address them in their revised manuscript. In relation to religious and social effects on opinion behavior in the U.S., I suggest that the authors check the outcomes in doi.org/10.1142/S0129183124501900 and in doi.org/10.1371/journal.pone.0331959, if relevant to their context in regulations.

It is important that the authors are diligent in their revisions, taking into account the changes suggested by the reviewers in their resubmission.

Please submit your revised manuscript by Mar 21 2026 11:59PM. If you will need more time than this to complete your revisions, please reply to this message or contact the journal office at plosone@plos.org. When you're ready to submit your revision, log on to https://www.editorialmanager.com/pone/ and select the 'Submissions Needing Revision' folder to locate your manuscript file.

Please include the following items when submitting your revised manuscript:

• A letter that responds to each point raised by the academic editor and reviewer(s). You should upload this letter as a separate file labeled 'Response to Reviewers'.

• A marked-up copy of your manuscript that highlights changes made to the original version. You should upload this as a separate file labeled 'Revised Manuscript with Track Changes'.

• An unmarked version of your revised paper without tracked changes. You should upload this as a separate file labeled 'Manuscript'.

If you would like to make changes to your financial disclosure, please include your updated statement in your cover letter. Guidelines for resubmitting your figure files are available below the reviewer comments at the end of this letter.

If applicable, we recommend that you deposit your laboratory protocols in protocols.io to enhance the reproducibility of your results. Protocols.io assigns your protocol its own identifier (DOI) so that it can be cited independently in the future. For instructions see: https://journals.plos.org/plosone/s/submission-guidelines#loc-laboratory-protocols. Additionally, PLOS ONE offers an option for publishing peer-reviewed Lab Protocol articles, which describe protocols hosted on protocols.io. Read more information on sharing protocols at https://plos.org/protocols?utm_medium=editorial-email&utm_source=authorletters&utm_campaign=protocols.

We look forward to receiving your revised manuscript.

Kind regards,

Omar El Deeb

Academic Editor

PLOS One

Journal Requirements:

When submitting your revision, we need you to address these additional requirements.

1. Please ensure that your manuscript meets PLOS ONE's style requirements, including those for file naming. The PLOS ONE style templates can be found at

https://journals.plos.org/plosone/s/file?id=wjVg/PLOSOne_formatting_sample_main_body.pdf and

https://journals.plos.org/plosone/s/file?id=ba62/PLOSOne_formatting_sample_title_authors_affiliations.pdf

� We thank the Editor for this clarification. We have carefully revised the manuscript to ensure full compliance with PLOS ONE’s formatting and style requirements. Specifically, we have adapted the structure, headings, references, and file naming conventions according to the official templates provided.

2. We noticed you have some minor occurrence of overlapping text with the following previous publication(s), which needs to be addressed:

- https://doi.org/10.1016/j.ssaho.2025.101529

In your revision ensure you cite all your sources (including your own works), and quote or rephrase any duplicated text outside the methods section. Further consideration is dependent on these concerns being addressed.

� We thank the Editor for raising this important issue. We have carefully reviewed the manuscript to identify and address any overlapping text with our previous publication.

3. Thank you for stating the following financial disclosure: [The author(s) disclosed receipt of the following financial support for the research, authorship, and/or publication of this article: This study was funded by the research fellowship “Catalina Ruiz,” provided by Gobierno de Canarias and the Agencia Canaria De Investigación Innovación Y Sociedad De La Información (ACIISI) and European Social Fund, through the Universidad de Las Palmas de Gran Canaria (Spain).].

Please state what role the funders took in the study. If the funders had no role, please state: ""The funders had no role in study design, data collection and analysis, decision to publish, or preparation of the manuscript.""

If this statement is not correct you must amend it as needed.

Please include this amended Role of Funder statement in your cover letter; we will change the online submission form on your behalf.

� We confirm that the funders had no role in the study design, data collection and analysis, decision to publish, or preparation of the manuscript. This statement has been included in the revised cover letter as requested, and we kindly ask the editorial office to update the submission system accordingly.

4. We note that the grant information you provided in the ‘Funding Information’ and ‘Financial Disclosure’ sections do not match.

When you resubmit, please ensure that you provide the correct grant numbers for the awards you received for your study in the ‘Funding Information’ section.

� We have carefully reviewed and corrected the funding information to ensure full consistency between the ‘Funding Information’ and ‘Financial Disclosure’ sections. The revised manuscript now includes the correct grant details and funding information across all sections.

5. Thank you for uploading your study's underlying data set. Unfortunately, the repository you have noted in your Data Availability statement does not qualify as an acceptable data repository according to PLOS's standards.

� We thank the Editor for highlighting this issue. We have updated the Data Availability Statement to include the corresponding DOI and access link to the repository. This ensures transparency, reproducibility, and full compliance with PLOS data requirements.

At this time, please upload the minimal data set necessary to replicate your study's findings to a stable, public repository (such as figshare or Dryad) and provide us with the relevant URLs, DOIs, or accession numbers that may be used to access these data. For a list of recommended repositories and additional information on PLOS standards for data deposition, please see https://journals.plos.org/plosone/s/recommended-repositories.

6. Please include captions for your Supporting Information files at the end of your manuscript, and update any in-text citations to match accordingly. Please see our Supporting Information guidelines for more information: http://journals.plos.org/plosone/s/supporting-information.

7. If the reviewer comments include a recommendation to cite specific previously published works, please review and evaluate these publications to determine whether they are relevant and should be cited. There is no requirement to cite these works unless the editor has indicated otherwise.

[Note: HTML markup is below. Please do not edit.]

� See below how we have responded to each of the comments made by the referees.

Reviewers' comments

Reviewer's Responses to Questions

Comments to the Author

1. Is the manuscript technically sound, and do the data support the conclusions?

The manuscript must describe a technically sound piece of scientific research with data that supports the conclusions. Experiments must have been conducted rigorously, with appropriate controls, replication, and sample sizes. The conclusions must be drawn appropriately based on the data presented.

Reviewer #1: Yes

Reviewer #2: No

________________________________________

2. Has the statistical analysis been performed appropriately and rigorously?

Reviewer #1: Yes

Reviewer #2: I Don't Know

________________________________________

3. Have the authors made all data underlying the findings in their manuscript fully available?

The PLOS Data policy requires authors to make all data underlying the findings described in their manuscript fully available without restriction, with rare exception (please refer to the Data Availability Statement in the manuscript PDF file). The data should be provided as part of the manuscript or its supporting information, or deposited to a public repository. For example, in addition to summary statistics, the data points behind means, medians and variance measures should be available. If there are restrictions on publicly sharing data—e.g. participant privacy or use of data from a third party—those must be specified.

Reviewer #1: Yes

Reviewer #2: Yes

________________________________________

4. Is the manuscript presented in an intelligible fashion and written in standard English?

PLOS ONE does not copyedit accepted manuscripts, so the language in submitted articles must be clear, correct, and unambiguous. Any typographical or grammatical errors should be corrected at revision, so please note any specific errors here.

Reviewer #1: Yes

Reviewer #2: Yes

________________________________________

5. Review Comments to the Author

Please use the space provided to explain your answers to the questions above. You may also include additional comments for the author, including concerns about dual publication, research ethics, or publication ethics. (Please upload your review as an attachment if it exceeds 20,000 characters)

Reviewer #1: Generally this is a well-written manuscript. You have answered the study question. The statistical analyses were adequate and your references were mainly up-to-date. Perhaps you can discuss about the limitations to the study before making a concluding remark.

We thank the reviewer for the positive and constructive feedback. In response to this suggestion, we have expanded the limitations of the study to provide a more balanced interpretation of the findings. Specifically, we have added a dedicated paragraph in the “Limitations of the study” section (after Discussions), where we discuss methodological constraints, including the use of a three-item proxy for perceived environmental regulation effects, the cross-sectional nature of the dataset, and the potential lack of generalisability beyond the U.S. context.

Reviewer #2: Some of the initial premises are unclear and appear to present an ideological stance and assumptions. Why is environmental regulation adequately represented by the proxy questions: (1) Much higher prices for fuel and electricity; (2) Gradual loss of individual freedoms; and (3) fewer jobs and declining pay in industries that depend on fossil fuels? These seem like issues which could / should be much more widely interpreted than environmental regulation. The author’s definition of Regulation needs to be given upfront.

� We thank the reviewer for this important and insightful comment. We acknowledge that the initial version of the manuscript did not sufficiently clarify the conceptualisation of environmental regulation within the scope of this study.

To address this, we have revised the Introduction to provide a clearer, more explicit definition of environmental regulation as a broad set of policies and institutional interventions that aim to mitigate environmental externalities. Importantly, we now emphasise that the study does not measure environmental regulation per se, but rather individuals’ perceptions of its potential socio-economic consequences.

Additionally, we have clarified that the three proxy items (energy prices, individual freedoms, and employment in fossil-fuel sectors) are derived from the Pew Research Centre survey and are interpreted as perceived outcomes associated with environmental regulation, rather than an exhaustive representation of the concept. This distinction is now explicitly stated to avoid any potential misinterpretation.

This continues throughout the paper. For example: “The environmental regulation to reduce greenhouse gas emissions will lead to higher electricity prices (Lee, 2022).” Firstly, higher electricity prices to who? Higher costs in generation are not necessarily transferred directly to the customer, that is a choice. Secondly, the seminal work on environmental regulation highlights that well designed regulation acts to reduce costs, and is designed to drive compliance, please see Porter, M. E., & Linde, C. V. D. (1995). Toward a new conception of the environment-competitiveness relationship. Journal of economic perspectives, 9(4), 97-118. There are multiple assessments of the application of the ‘Porter Hypothesis’ but this gets only a tangential mention in the paper and is not explained.

� We agree that the relationship between environmental regulation and electricity prices is complex and context-dependent. In response, we have revised the Literature Review to clarify that increases in electricity prices are not automatic or universal outcomes of environmental regulation, but rather depend on market structures, policy design, and regulatory choices. We now explicitly acknowledge that cost pass-through to consumers is not guaranteed.

Furthermore, we have expanded the discussion of the Porter Hypothesis by incorporating a more detailed explanation of how well-designed environmental regulation can foster innovation, improve efficiency, and potentially reduce long-term costs. This theoretical perspective is now more clearly integrated into the manuscript, providing a more balanced and nuanced interpretation of the economic impacts of environmental regulation.

There are some very strong statements made with very limited justification. For example: “Environmental regulations have caused an important decline in the coal industry worldwide, leading to job losses in various industry sectors, including mining, transportation, and power generation.” I don’t think you can attribute the shifts in the coal industry to environmental regulation alone. In fact, a very quick check highlights that natural gas may have as significant an impact as environmental regulation, for example: Houser, T., Bordoff, J., & Marsters, P. (2017). Can coal make a comeback?. New York, NY: Columbia Center on Global Energy Policy.

� We agree that attributing the decline of the coal industry solely to environmental regulation is an oversimplification. We now explicitly acknowledge that the decline of the coal industry is driven by multiple factors, including technological change, market dynamics, and, in particular, the increasing competitiveness of natural gas, as highlighted by Houser et al. (2017).

The authors say that “We could not find any scale developed to measure the environmental regulation effects perception”. There is a well-established literature base on the perception of regulation. See Valerie Braithwaite’s work, for example: Braithwaite, V. (2003). Dancing with tax authorities: Motivational postures and non-compliant actions. Taxing democracy, 3, 15-39. Or Braithwaite, V. (2017). Closing the gap between regulation and the community. Regulatory theory: Foundations and applications, 59(2), 25-42. It isn’t clear why the authors would not use these as a starting point. In addition, the perceived and actual effects of environmental regulation, for example on competitiveness, cost of implementation, etc. Have been well documented and discussed across multiple papers, to pick out one example: López-Gamero, M. D., Molina-Azorín, J. F., & Claver-Cortés, E. (2010). The potential of environmental regulation to change managerial per

Attachment Submitted filename: Response to Referees Reports.docx

# Peer review: Decision Letter 1

**DOI:** 10.1371/journal.pone.0334868.r003

**Authors:** Omar El Deeb

21 Apr 2026

PONE-D-25-51401R1

A Fuzzy Clustering Approach to Understanding Religious and Socioeconomic Determinants of Public Perceptions of Environmental Regulation in the United States

PLOS One

Dear Dr.  Indelicato,

Thank you for submitting your manuscript to PLOS ONE. After careful consideration, we feel that it has merit but does not fully meet PLOS ONE’s publication criteria as it currently stands. Therefore, we invite you to submit a revised version of the manuscript that addresses the points raised during the review process.

Please submit your revised manuscript by Jun 05 2026 11:59PM. If you will need more time than this to complete your revisions, please reply to this message or contact the journal office at plosone@plos.org. When you're ready to submit your revision, log on to https://www.editorialmanager.com/pone/ and select the 'Submissions Needing Revision' folder to locate your manuscript file.

Please include the following items when submitting your revised manuscript:

- A letter that responds to each point raised by the academic editor and reviewer(s). You should upload this letter as a separate file labeled 'Response to Reviewers'.
- A marked-up copy of your manuscript that highlights changes made to the original version. You should upload this as a separate file labeled 'Revised Manuscript with Track Changes'.
- An unmarked version of your revised paper without tracked changes. You should upload this as a separate file labeled 'Manuscript'.

If you would like to make changes to your financial disclosure, please include your updated statement in your cover letter. Guidelines for resubmitting your figure files are available below the reviewer comments at the end of this letter.

If applicable, we recommend that you deposit your laboratory protocols in protocols.io to enhance the reproducibility of your results. Protocols.io assigns your protocol its own identifier (DOI) so that it can be cited independently in the future. For instructions see: https://journals.plos.org/plosone/s/submission-guidelines#loc-laboratory-protocols. Additionally, PLOS ONE offers an option for publishing peer-reviewed Lab Protocol articles, which describe protocols hosted on protocols.io. Read more information on sharing protocols at https://plos.org/protocols?utm_medium=editorial-email&utm_source=authorletters&utm_campaign=protocols.

As the corresponding author, your ORCID iD is verified in the submission system and will appear in the published article. PLOS supports the use of ORCID, and we encourage all coauthors to register for an ORCID iD and use it as well. Please encourage your coauthors to verify their ORCID iD within the submission system before final acceptance, as unverified ORCID iDs will not appear in the published article. *Only*  the individual author can complete the verification step; PLOS staff *cannot*  verify ORCID iDs on behalf of authors.

We look forward to receiving your revised manuscript.

Kind regards,

Omar El Deeb

Academic Editor

PLOS One

Journal Requirements:

1. If the reviewer comments include a recommendation to cite specific previously published works, please review and evaluate these publications to determine whether they are relevant and should be cited. There is no requirement to cite these works unless the editor has indicated otherwise.

2. Please review your reference list to ensure that it is complete and correct. If you have cited papers that have been retracted, please include the rationale for doing so in the manuscript text, or remove these references and replace them with relevant current references. Any changes to the reference list should be mentioned in the rebuttal letter that accompanies your revised manuscript. If you need to cite a retracted article, indicate the article’s retracted status in the References list and also include a citation and full reference for the retraction notice.

[Note: HTML markup is below. Please do not edit.]

Reviewer's Responses to Questions

**Comments to the Author**

1. If the authors have adequately addressed your comments raised in a previous round of review and you feel that this manuscript is now acceptable for publication, you may indicate that here to bypass the “Comments to the Author” section, enter your conflict of interest statement in the “Confidential to Editor” section, and submit your "Accept" recommendation.

Reviewer #3: (No Response)

**********

2. Is the manuscript technically sound, and do the data support the conclusions?

The manuscript must describe a technically sound piece of scientific research with data that supports the conclusions. Experiments must have been conducted rigorously, with appropriate controls, replication, and sample sizes. The conclusions must be drawn appropriately based on the data presented.

Reviewer #3: Yes

**********

3. Has the statistical analysis been performed appropriately and rigorously?

Reviewer #3: Yes

**********

4. Have the authors made all data underlying the findings in their manuscript fully available?

The PLOS Data policy requires authors to make all data underlying the findings described in their manuscript fully available without restriction, with rare exception (please refer to the Data Availability Statement in the manuscript PDF file). The data should be provided as part of the manuscript or its supporting information, or deposited to a public repository. For example, in addition to summary statistics, the data points behind means, medians and variance measures should be available. If there are restrictions on publicly sharing data—e.g. participant privacy or use of data from a third party—those must be specified.

Reviewer #3: No

**********

5. Is the manuscript presented in an intelligible fashion and written in standard English?

PLOS ONE does not copyedit accepted manuscripts, so the language in submitted articles must be clear, correct, and unambiguous. Any typographical or grammatical errors should be corrected at revision, so please note any specific errors here.

Reviewer #3: Yes

**********

6. Review Comments to the Author

Please use the space provided to explain your answers to the questions above. You may also include additional comments for the author, including concerns about dual publication, research ethics, or publication ethics. (Please upload your review as an attachment if it exceeds 20,000 characters)

Reviewer #3: A Fuzzy Clustering Approach to Understanding Religious and Socioeconomic Determinants of Public Perceptions of Environmental Regulation in the United States

Reviewer Report:

1. The manuscript identifies three perceived outcomes of environmental regulation:

(i) higher fuel and electricity prices: This outcome can plausibly be linked to environmental regulation through mechanisms such as carbon pricing, emissions standards, and compliance costs imposed on energy producers. However, price is determined by the general supply-demand Dynamics.

(ii) loss of individual freedoms: This is not inherently a direct consequence of environmental regulation but rather reflects public perception of regulatory overreach. The authors should clarify that this dimension captures subjective interpretations (e.g., restrictions on driving a 4000 cc) rather than objective regulatory outcomes.

(iii) reduced employment in fossil-fuel-based industries: While job losses may occur in carbon-intensive sectors due to environmental policies, attributing them solely to regulation is overly simplistic. Structural changes such as automation and market transitions toward renewable energy also play a role.

Without clarifications, the constructs appear theoretically weak. It should be strictly stressed that these are perceptions, thoughts of US consumers. The authors should reframe these variables explicitly as perceived consequences rather than objective effects. The theoretical linkage between these outcomes and environmental regulation is currently underdeveloped and, in parts, conceptually inconsistent. (Still)

2. Typographical Error

The phrase “Mormon (Church of Jesus Christ” “)” contains a formatting error (missing closing parenthesis or incorrect punctuation).

3. Inconsistency in Grouping Religious Categories

There is a lack of consistency between the table presentation and the textual interpretation:

In Table 2-3, religious groups such as Mormons and Protestants are presented as distinct categories, yet they are combined in the text.

Similarly, Atheists, Muslims, and Hindus are grouped together in the text despite being separate categories in the table.

Provide a clear rationale for aggregating categories (e.g., theoretical similarity, cluster analysis results, or sample size limitations). Ensure consistency between tables and narrative.

If grouping is necessary, consider reporting both disaggregated and aggregated results for transparency.

**********

7. PLOS authors have the option to publish the peer review history of their article (what does this mean?). If published, this will include your full peer review and any attached files.

If you choose “no”, your identity will remain anonymous but your review may still be made public.

**Do you want your identity to be public for this peer review?**  For information about this choice, including consent withdrawal, please see our Privacy Policy.

Reviewer #3: No

**********

[NOTE: If reviewer comments were submitted as an attachment file, they will be attached to this email and accessible via the submission site. Please log into your account, locate the manuscript record, and check for the action link "View Attachments". If this link does not appear, there are no attachment files.]

To ensure your figures meet our technical requirements, please review our figure guidelines: https://journals.plos.org/plosone/s/figures

You may also use PLOS’s free figure tool, NAAS, to help you prepare publication quality figures: https://journals.plos.org/plosone/s/figures#loc-tools-for-figure-preparation.

NAAS will assess whether your figures meet our technical requirements by comparing each figure against our figure specifications.

# Author comment: Author response to Decision Letter 2

**DOI:** 10.1371/journal.pone.0334868.r004

26 May 2026

PONE-D-25-51401R1

A Fuzzy Clustering Approach to Understanding Religious and Socioeconomic Determinants of Public Perceptions of Environmental Regulation in the United States

PLOS One

The authors acknowledge the editor and reviewers for their comments, which improved the paper's presentation. This document outlines our responses to the editor and reviewers’ comments. Our responses are italicized and indicated by a “�” sign.

Dear Dr. ,

Thank you for submitting your manuscript to PLOS ONE. After careful consideration, we feel that it has merit but does not fully meet PLOS ONE’s publication criteria as it currently stands. Therefore, we invite you to submit a revised version of the manuscript that addresses the points raised during the review process.

Please submit your revised manuscript by Jun 05 2026 11:59PM. If you will need more time than this to complete your revisions, please reply to this message or contact the journal office at plosone@plos.org. When you're ready to submit your revision, log on to https://www.editorialmanager.com/pone/ and select the 'Submissions Needing Revision' folder to locate your manuscript file.

Please include the following items when submitting your revised manuscript:

• A letter that responds to each point raised by the academic editor and reviewer(s). You should upload this letter as a separate file labeled 'Response to Reviewers'.

• A marked-up copy of your manuscript that highlights changes made to the original version. You should upload this as a separate file labeled 'Revised Manuscript with Track Changes'.

• An unmarked version of your revised paper without tracked changes. You should upload this as a separate file labeled 'Manuscript'.

If you would like to make changes to your financial disclosure, please include your updated statement in your cover letter. Guidelines for resubmitting your figure files are available below the reviewer comments at the end of this letter.

If applicable, we recommend that you deposit your laboratory protocols in protocols.io to enhance the reproducibility of your results. Protocols.io assigns your protocol its own identifier (DOI) so that it can be cited independently in the future. For instructions see: https://journals.plos.org/plosone/s/submission-guidelines#loc-laboratory-protocols.

Additionally, PLOS ONE offers an option for publishing peer-reviewed Lab Protocol articles, which describe protocols hosted on protocols.io. Read more information on sharing protocols at https://plos.org/protocols?utm_medium=editorial-email&utm_source=authorletters&utm_campaign=protocols.

As the corresponding author, your ORCID iD is verified in the submission system and will appear in the published article. PLOS supports the use of ORCID, and we encourage all coauthors to register for an ORCID iD and use it as well. Please encourage your coauthors to verify their ORCID iD within the submission system before final acceptance, as unverified ORCID iDs will not appear in the published article. Only the individual author can complete the verification step; PLOS staff cannot verify ORCID iDs on behalf of authors.

We look forward to receiving your revised manuscript.

Kind regards,

Omar El Deeb

Academic Editor

PLOS One

Journal Requirements:

1. If the reviewer comments include a recommendation to cite specific previously published works, please review and evaluate these publications to determine whether they are relevant and should be cited. There is no requirement to cite these works unless the editor has indicated otherwise.

We thank the Academic Editor for this suggestion. We have carefully considered the reviewer’s comments and the literature. Where relevant, we have added references that support the theoretical framing of the revised manuscript.

2. Please review your reference list to ensure that it is complete and correct. If you have cited papers that have been retracted, please include the rationale for doing so in the manuscript text, or remove these references and replace them with relevant current references. Any changes to the reference list should be mentioned in the rebuttal letter that accompanies your revised manuscript. If you need to cite a retracted article, indicate the article’s retracted status in the References list and also include a citation and full reference for the retraction notice.

�The reference list has been checked thoroughly to ensure all entries are complete, correct and comply with the journal’s requirements. We also checked the cited literature for retraction notices and found no retracted articles among the references used in the revised manuscript.

[Note: HTML markup is below. Please do not edit.]

Reviewer's Responses to Questions

Comments to the Author

1. If the authors have adequately addressed your comments raised in a previous round of review and you feel that this manuscript is now acceptable for publication, you may indicate that here to bypass the “Comments to the Author” section, enter your conflict of interest statement in the “Confidential to Editor” section, and submit your "Accept" recommendation.

Reviewer #3: (No Response)

________________________________________

2. Is the manuscript technically sound, and do the data support the conclusions?

The manuscript must describe a technically sound piece of scientific research with data that supports the conclusions. Experiments must have been conducted rigorously, with appropriate controls, replication, and sample sizes. The conclusions must be drawn appropriately based on the data presented.

Reviewer #3: Yes

________________________________________

3. Has the statistical analysis been performed appropriately and rigorously?

Reviewer #3: Yes

________________________________________

4. Have the authors made all data underlying the findings in their manuscript fully available?

The PLOS Data policy requires authors to make all data underlying the findings described in their manuscript fully available without restriction, with rare exception (please refer to the Data Availability Statement in the manuscript PDF file). The data should be provided as part of the manuscript or its supporting information, or deposited to a public repository. For example, in addition to summary statistics, the data points behind means, medians and variance measures should be available. If there are restrictions on publicly sharing data—e.g. participant privacy or use of data from a third party—those must be specified.

Reviewer #3: No

________________________________________

5. Is the manuscript presented in an intelligible fashion and written in standard English?

PLOS ONE does not copyedit accepted manuscripts, so the language in submitted articles must be clear, correct, and unambiguous. Any typographical or grammatical errors should be corrected at revision, so please note any specific errors here.

Reviewer #3: Yes

________________________________________

6. Review Comments to the Author

Please use the space provided to explain your answers to the questions above. You may also include additional comments for the author, including concerns about dual publication, research ethics, or publication ethics. (Please upload your review as an attachment if it exceeds 20,000 characters)

Reviewer #3: A Fuzzy Clustering Approach to Understanding Religious and Socioeconomic Determinants of Public Perceptions of Environmental Regulation in the United States

Reviewer Report:

1. The manuscript identifies three perceived outcomes of environmental regulation:

(i) higher fuel and electricity prices: This outcome can plausibly be linked to environmental regulation through mechanisms such as carbon pricing, emissions standards, and compliance costs imposed on energy producers. However, price is determined by the general supply-demand Dynamics.

�We thank the reviewer for this useful comment. We have clarified in the Literature Review (Environmental Regulation Impacts) that higher fuel and electricity prices are a perceived consequence of environmental regulation rather than an objective price effect. We now also acknowledge that energy prices are shaped by supply-demand dynamics, market volatility, infrastructure constraints, geopolitical shocks and policy design.

(ii) loss of individual freedoms: This is not inherently a direct consequence of environmental regulation but rather reflects public perception of regulatory overreach. The authors should clarify that this dimension captures subjective interpretations (e.g., restrictions on driving a 4000 cc) rather than objective regulatory outcomes.

We thank the reviewer for this important clarification. We have revised the Literature Review (Environmental Regulation Effects Surveys). Now, it is specified that this item captures respondents’ subjective perception of regulatory overreach, rather than a loss of individual freedoms that could be objectively observed.

(iii) reduced employment in fossil-fuel-based industries: While job losses may occur in carbon-intensive sectors due to environmental policies, attributing them solely to regulation is overly simplistic. Structural changes such as automation and market transitions toward renewable energy also play a role.

We agree with the reviewer. As we have reviewed in the Literature Review (Environmental Regulation Impacts section), the decline in employment in fossil-fuel-dependent industries cannot be attributed solely to environmental regulation. The revised manuscript now recognises the roles of automation, market restructuring, and the transition to renewable energy.

Without clarifications, the constructs appear theoretically weak. It should be strictly stressed that these are perceptions, thoughts of US consumers. The authors should reframe these variables explicitly as perceived consequences rather than objective effects. The theoretical linkage between these outcomes and environmental regulation is currently underdeveloped and, in parts, conceptually inconsistent. (Still)

�We thank the reviewer for this valuable observation. We have revised the Abstract, Introduction, Variables section, Methodology, Results, and Discussions to clarify that the three items are indicators of perception. The AP-ERE index is explicitly described as a measure of respondents' perceived likelihood of potential consequences, rather than as evidence of objective or causal regulatory effects.

2. Typographical Error

The phrase “Mormon (Church of Jesus Christ” “)” contains a formatting error (missing closing parenthesis or incorrect punctuation).

�We thank the reviewer for pointing this out. We have corrected the formatting error in Tables 2 and 3 and now report the category consistently as “Mormon/Latter-day Saint” throughout the manuscript.

3. Inconsistency in Grouping Religious Categories

There is a lack of consistency between the table presentation and the textual interpretation:

In Table 2-3, religious groups such as Mormons and Protestants are presented as distinct categories, yet they are combined in the text.

Similarly, Atheists, Muslims, and Hindus are grouped together in the text despite being separate categories in the table.

Provide a clear rationale for aggregating categories (e.g., theoretical similarity, cluster analysis results, or sample size limitations). Ensure consistency between tables and narrative.

If grouping is necessary, consider reporting both disaggregated and aggregated results for transparency.

We thank the reviewer for this constructive comment. As we explained in the Variables section, religious categories were analysed separately rather than formally aggregated. We also revised the Results and Discussion sections to align with Tables 2 and 3. Some categories are mentioned together for interpretative purposes only, as they exhibit similar descriptive patterns.

________________________________________

7. PLOS authors have the option to publish the peer review history of their article (what does this mean?). If published, this will include your full peer review and any attached files.

If you choose “no”, your identity will remain anonymous but your review may still be made public.

Do you want your identity to be public for this peer review? For information about this choice, including consent withdrawal, please see our Privacy Policy.

Reviewer #3: No

________________________________________

[NOTE: If reviewer comments were submitted as an attachment file, they will be attached to this email and accessible via the submission site. Please log into your account, locate the manuscript record, and check for the action link "View Attachments". If this link does not appear, there are no attachment files.]

To ensure your figures meet our technical requirements, please review our figure guidelines: https://journals.plos.org/plosone/s/figures

You may also use PLOS’s free figure tool, NAAS, to help you prepare publication quality figures: https://journals.plos.org/plosone/s/figures#loc-tools-for-figure-preparation.

NAAS will assess whether your figures meet our technical requirements by comparing each figure against our figure specifications.

We are grateful to the Academic Editor and Reviewers for their careful reading of the manuscript and constructive comments. Their comments were useful in clarifying the study's conceptual framing, strengthening the interpretation of the perception-based indicators, and improving the consistency of the presentation. We think that the revised manuscript is now clearer, more precise and better adapted to the aims and standards of PLOS ONE.

Attachment Submitted filename: Response_to_Referees_Reports_auresp_2.docx

# Editor report: Decision Letter 2

**DOI:** 10.1371/journal.pone.0334868.r005

**Authors:** Omar El Deeb

18 Jun 2026

A Fuzzy Clustering Approach to Understanding Religious and Socioeconomic Determinants of Public Perceptions of Environmental Regulation in the United States

PONE-D-25-51401R2

Dear Dr. Indelicato,

We’re pleased to inform you that your manuscript has been judged scientifically suitable for publication and will be formally accepted for publication once it meets all outstanding technical requirements.

Within one week, you’ll receive an e-mail detailing the required amendments. When these have been addressed, you’ll receive a formal acceptance letter and your manuscript will be scheduled for publication.

An invoice will be generated when your article is formally accepted. Please note, if your institution has a publishing partnership with PLOS and your article meets the relevant criteria, all or part of your publication costs will be covered. Please make sure your user information is up-to-date by logging into Editorial Manager at Editorial Manager® and clicking the ‘Update My Information' link at the top of the page. For questions related to billing, please contact billing support.

If your institution or institutions have a press office, please notify them about your upcoming paper to help maximize its impact. If they’ll be preparing press materials, please inform our press team as soon as possible -- no later than 48 hours after receiving the formal acceptance. Your manuscript will remain under strict press embargo until 2 pm Eastern Time on the date of publication. For more information, please contact onepress@plos.org.

Kind regards,

Omar El Deeb

Academic Editor

PLOS One

Additional Editor Comments (optional):

Reviewers' comments:
